# Integrated genomics with refined cell-of-origin subtyping distinguishes subtype-specific mechanisms of treatment resistance and relapse in diffuse large B-cell lymphoma

**DOI:** 10.1038/s41408-025-01326-5

**Published:** 2025-07-12

**Authors:** Janek S. Walker, Kerstin Wenzl, Joseph P. Novak, Matthew E. Stokes, Melissa A. Hopper, Abigail R. Dropik, Miranda S. Siminski, Allison M. Bock, Vivekananda Sarangi, Maria Ortiz, Nicholas Stong, C. Chris Huang, Matthew J. Maurer, Brian K. Link, Stephen M. Ansell, Thomas M. Habermann, Thomas E. Witzig, Rebecca L. King, Grzegorz Nowakowski, James R. Cerhan, Anita K. Gandhi, Anne J. Novak

**Affiliations:** 1 Division of Hematology, Mayo Clinic, Rochester, MN USA; 2 Informatics and Predictive Sciences, Bristol Myers Squibb, Summit, NJ USA; 3 Division of Hematology and Hematologic Malignancies, Huntsman Cancer Institute/University of Utah, Salt Lake City, UT USA; 4 Department of Quantitative Health Sciences Research, Mayo Clinic, Rochester, MN USA; 5 Informatics and Predictive Sciences, Celgene Institute for Translational Research Europe (CITRE), Seville, Spain; 6 Translational Medicine Hematology, Bristol Myers Squibb, Summit, NJ USA; 7 Division of Hematology, University of Iowa, Iowa City, IA USA; 8 Department of Laboratory Medicine and Pathology, Mayo Clinic, Rochester, MN USA

**Keywords:** B-cell lymphoma, Cancer genomics, Oncogenes, Genetics research

## Abstract

Up to 40% of diffuse large B-cell lymphoma (DLBCL) patients do not experience a durable response to frontline immunochemotherapy, and prospective identification of high-risk cases that may benefit from personalized therapeutic management remains an unmet need. Molecular phenotyping techniques have established a landscape of genomic variants in diagnostic DLBCL; however, these have not yet been applied in large-scale studies of relapsed/refractory DLBCL, resulting in incomplete characterization of mechanisms driving tumor progression and treatment resistance. Here, we performed an integrated multiomic analysis on 228 relapsed/refractory DLBCL samples, including 24 with serial biopsies. Refined cell-of-origin subtyping identified patients harboring GCB and DZsig+ relapsed/refractory tumors in cases with primary refractory disease with remarkably poor outcomes, and comparative analysis of genomic features between relapsed and diagnostic samples identified subtype-specific mechanisms of therapeutic resistance driven by frequent alteration to *MYC*, *BCL2*, *BCL6*, and *TP53* among additional strong lymphoma driver genes. Tumor evolution dynamics suggest innate mechanisms of chemoresistance are present in many DLBCL tumors at diagnosis, and that relapsed/refractory tumors are primarily comprised of a homogenous clonal expansion with reduced tumor microenvironment activity. Adaptation of personalized therapeutic strategies targeting DLBCL subtype-specific resistance mechanisms should be considered to benefit these high-risk populations.

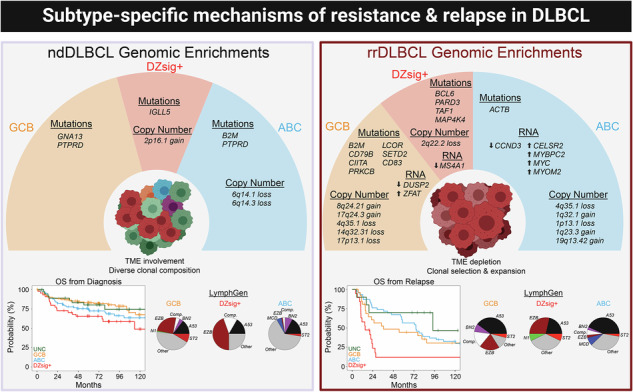

## Introduction

Diffuse large B-cell lymphoma (DLBCL) is a highly heterogeneous lymphoma representing ~30% of non-Hodgkin lymphoma cases worldwide. Patient outcomes are favorable, with more than 65% of patients experiencing durable remission following standard-of-care rituximab with cyclophosphamide, vincristine, doxorubicin, and prednisone (R-CHOP) or polatuzumab vedotin (pola-R-CHP) immunochemotherapy [1–5]. In contrast, 30–40% of DLBCL patients will relapse following an initial response, with ~10% of cases exhibiting primary refractory disease [6]. Outcomes for relapsed/refractory DLBCL (rrDLBCL) cases remain poor, particularly for those with tumor progression within 24 months from diagnosis [7, 8]. rrDLBCL cases may be eligible for autologous stem-cell transplantation (ACST), chimeric antigen-receptor T-cell (CAR-T), or bispecific T-cell engager (epcoritamab, glofitamab) therapy, however complete responses in this setting remain below 50%, representing a major clinical challenge with significant unmet needs [4, 9–11].

Recent efforts to define the high degree of complexity among DLBCL tumors have been dominated by advancements in high-throughput systems facilitating the identification of molecular subtypes with distinct pathogenic mechanisms that respectively associate with clinical features and risk factors. [12–17] Adoption of these classifications by research groups has expanded comprehension of genomic landscapes underlying DLBCL tumors at diagnosis [18–21], and recent studies have begun to utilize these techniques to evaluate rrDLBCL. These studies have described disruption to apoptotic, senescence, and NF-κB signaling pathways as factors driving therapeutic resistance, and provide evidence suggesting distinct tumor evolutionary dynamics in early vs late relapse cases [8, 22–26]; overall broadening the scope of understanding of rrDLBCL tumor biology. However, the studies to date remain restricted by sample size with limited evaluation of subtype-specific disease biology, insufficient to comprehensively describe resistance mechanisms and translate to benefit high-risk patients.

To expand upon the current etiology and driving mechanisms of resistance in DLBCL, the study presented herein describes the largest multiomic analysis in rrDLBCL tumor samples to date, utilizing clinical and biologic variables from 228 rrDLBCL and 444 newly diagnosed DLBCL (ndDLBCL). We report that the genomic landscape among rrDLBCL tumors collectively shares considerable overlap with diagnostic DLBCL, validating literature describing very few genomic variants enriched in rrDLBCL vs ndDLBCL. Alternatively, leveraging refined cell-of-origin (COO) subtyping including the dark-zone signature positive (DZsig+) classification [17] to facilitate comparative analysis of genomic and transcriptomic alterations within tumor sub-groups, we identify GCB and DZsig+ subtype rrDLBCL tumors more frequently observed in aggressive, primary treatment refractory, cases with differential underlying biology defined by enrichment for somatic variants and copy number alterations affecting *MYC*, *BCL2*, *BCL6* and *TP53* among additional strong lymphoma driver genes. Clonal evolution analysis in rrDLBCL suggests the genomic landscape driving treatment resistance is strongly present at diagnosis, and exceedingly evident in cases of primary refractory disease. Herein, we explore subtype-specific molecular features in rrDLBCL tumors and establish a framework to identify potential high-risk cases and proactively strategize clinical management beyond frontline treatment.

## Materials/subjects and methods

### Study populations

Participants were not prospectively identified for this study. All samples had a diagnosis of DLBCL. All patients provided written informed consent at study enrollment, including use of clinical samples in accordance with the Declaration of Helsinki, and approved by the institutional review board (IRB) at study centers. We utilized clinical and high-throughput sequencing data from 228 relapsed/refractory and 444 diagnostic DLBCL tumors. [27] rrDLBCL tumors obtained from *n* = 85 participants enrolled in the Mayo Clinic/University of Iowa Lymphoma Molecular Epidemiology Resource (MER) program; *n* = 73 in the Mayo Clinic Lymphoma Biobank; and *n* = 70 rrDLBCL collected as part of the clinical study NCT01421524 (Table 1). Similar clinical characteristics were observed in patients across the study sources (Supplemental Table 1). A majority of rrDLBCL patients (72.8%) received frontline R-CHOP (Supplemental Table 2).

**Table 1:** Patient characteristics.

Patient characteristics	All eligible rrDLBCL	Patient characteristics	All eligible rrDLBCL
	(*N* = 228)		(*N* = 228)
Age at diagnosis		Dark-zone signature, *n* (%)	
Mean (SD)	62.4 (13)	ABC	68 (46.6%)
Median	64.0	GCB	39 (26.7%)
IQR	55.0, 72.0	DZsig+	24 (16.4%)
Range	24.0, 91.0	Unclassified	15 (10.3%)
Age Group, *n* (%)		EFS24, *n* (%)	
≤60	91 (41.0%)	Achieved	36 (23.8%)
>60	131 (59.0%)	Failed	115 (76.2%)
Sex, *n* (%)		Last follow-up, *n* (%)	
Male	142 (64.0%)	Alive	52 (28.0%)
Female	80 (36.0%)	Dead	134 (72.0%)
PS Group, *n* (%)		Primary COD, *n* (%)	
<2	120 (85.7%)	Disease	88 (88.0%)
≥2	20 (14.3%)	Therapy	5 (5.0%)
Ann Arbor Stage, *n* (%)		Other	7 (7.0%)
I–II	37 (24.2%)	Dx to relapse sample (Mo)	
III–IV	116 (75.8%)	Mean (SD)	24.9 (28)
aaIPI, *n* (%)		Median	13.8
0	33 (15.1%)	IQR	7.9, 29.0
1	87 (39.7%)	Range	1.64, 149.8
2	82 (37.4%)	Relapse to last follow-up	
3	17 (7.8%)	Mean (SD)	39.9 (53)
Extranodal sites, *n* (%)		Median	10.2
0–1 extranodal sites	122 (77.2%)	IQR	3.45, 65.0
2 or more extranodal sites	36 (22.8%)	Range	0.03, 210.1
Bulky disease, *n* (%)		Sample Type, *n* (%)	
Yes	13 (8.4%)	R1	96 (60.8%)
No	142 (91.6%)	R2	36 (22.8%)
Bone marrow involv., *n* (%)		R3	17 (10.8%
Yes	33 (23.2%)	R4	4 (2.5%)
None	109 (76.8%)	R5	2 (1.3%)
B symptoms, *n* (%)		R6	1 (0.6%)
Yes	44 (30.1%)	R7	1 (0.6%)
No	102 (69.9%)	R10	1 (0.6%)
LDH group, *n* (%)			
Elevated	110 (67.5%)		
Not elevated	53 (32.5%)		

Clinical and HTS data from ndDLBCL tumors were obtained from the MER or trial NCT00670358 [28], as described in Wenzl et al. [27]. A total of 693 DLBCL tumor samples were evaluated for comparative analysis of features between diagnostic and relapsed DLBCL, *n* = 444 ndDLBCL and *n* = 249 rrDLBCL (Supplemental Fig. 1). In the case where both relapsed and diagnostic tumor samples were collected from the same patient, that sample was removed from the ndDLBCL cohort (*n* = 24). Comparative analysis between ndDLBCL was conducted using the first available rrDLBCL sample for a given patient, removing *n* = 21 serial samples from the rrDLBCL cohort. Unless otherwise stated, genetic and clinical variables were evaluated between *n* = 420 ndDLBCL and *n* = 228 rrDLBCL cases. The differences in clinical characteristics in rrDLBCL and ndDLBCL were as expected (Supplemental Table 3). Clinical characteristics in rrDLBCL and ndDLBCL cohorts were largely representative of a tertiary care center and similar to population-based cohorts from studies in the USA, Canada, Denmark, and Sweden [8, 29–31]. The median age at relapse among rrDLBCL patients was 64.0 years [range 24–91], and 64% were male. The median time from initial DLBCL diagnosis to relapse/refractory sample acquisition was 13.8 months (range 1.64–149.8), with 76% of patients failing to achieve event-free status at 24 months (EFS24; Table 1, Supplemental Fig. 2A). Patients failing to achieve event-free status at 24 months demonstrated inferior overall survival (OS, measured from the time of rr sample collection to last follow-up; Log-rank *p* = 0.03; Supplemental Fig. 2B).

### DNA and RNA sequencing and analysis

The mutational profiles of *n* = 204 rrDLBCL tumors were assessed by whole-exome (WES) or whole-genome sequencing (WGS; Supplemental Fig. 2C). Paired germline DNA (from blood PBMC) was available for *n* = 143 rrDLBCL samples. All samples used for DNA and RNA extraction were reviewed by a Mayo Clinic hematopathologist prior to sectioning. All tumor DNA was extracted from formalin-fixed, paraffin-embedded (FFPE) tissue sections, and WES was performed at Expression Analysis using the Agilent SureSelect XT All Exon v6 + UTR kit, and sequencing was carried out on an Illumina NovaSeq, 100 × 2 paired-end reads. Average median sequencing coverage was reported at 78.4× across samples analyzed by WES and 38.2× across samples analyzed by WGS (Supplemental Table 4). Similar mutational profiles were observed in tumors between study and sequencing sources (Supplemental Fig. 2D, E). ndDLBCL samples were solely analyzed by WES using identical methods as above [27] with a median target coverage distribution similar to that observed in rrDLBCL samples (average median coverage = 80.7×). All ndDLBCL samples analyzed by WES had matched constitutional DNA sequencing. Gene mutations (*n* = 292) and genomic regions (*n* = 45) highlighted for enrichment analyses have been previously reported with impact on lymphoma biology (Supplemental Tables 5 and 6) [15, 32–38]. Frequency of mutations in genes with established potential in DLBCL in ndDLBCL and rrDLBCL cohorts was similar to three ndDLBCL validation cohorts: NCI (*n* = 489) [33], BCCA (*n* = 153) [21], and MSK-IMPACT (*n* = 220 [39]; Supplemental Fig. 2F). CNV analysis was conducted on *n* = 365 ndDLBCL and *n* = 132 rrDLBCL cases, all with matched constitutional DNA sequencing. High concordance in CNV frequency was observed in rrDLBCL samples sequenced by WES (*n* = 62) or WGS (*n* = 70) (Pearson’s *r* = 0.82, CI = [0.68–0.90], *p* < 0.001; Supplemental Fig. 2G).

RNA for RNA sequencing was extracted from FFPE tissue sections, and sequencing was performed at Expression Analysis, Inc. using the Illumina TruSeq RNA Exome Kit (Illumina) for library preparation, sequencing platform HiSeq 4000, 100 × 2 paired-end reads. Paired-end fastq files were processed on a cloud-based platform at Bristol Myers Squibb. Similar transcript profiles were observed in patient samples from all sample sources (Supplemental Fig. 3A–D). Cell of origin (COO) in ndDLBCL samples was obtained from Wenzl et al. [27], determined by the Lymph2Cx assay (NanoString; *n* = 224) [40] or from RNA-Seq data (*n* = 81) using the method described by Reddy et al. [32]. For rrDLBCL samples, COO status was assigned from RNA-Seq data according to the Reddy method and was shown to have excellent concordance (Pearson’s *r* = 0.95, CI = [0.93–0.96], *p* < 0.001) with the gneSeqCOO method [41] (Supplemental Fig. 3E). “Refined COO” subtype was assigned by applying the DHIT/DZsig method [17, 42]. Full sequencing and analysis details are available in the supplemental methods.

## Results

### Landscape of somatic mutations and structural variants in rrDLBCL

**Fig. 1:**
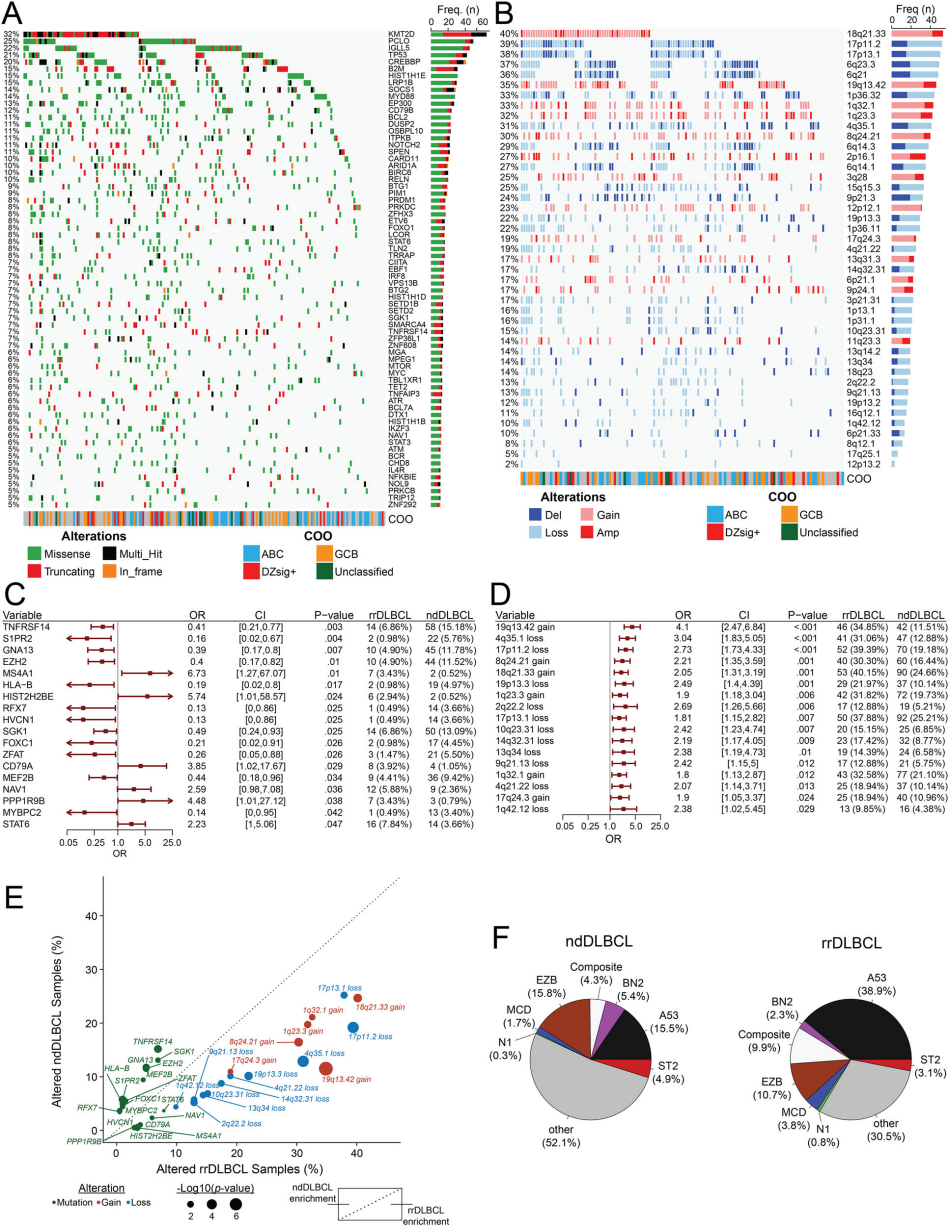
Genomic landscape and enrichment of genetic variants in rrDLBCL tumors. **A** Oncoplot representing recurrent non-silent somatic single variants or insertions/deletions in rrDLBCL tumor samples (*n* = 204). Predicted functional consequence represented by color (missense, green; truncating, red; in-frame, orange; multi-hit, black). Bar plot (right) represents the sum of samples with a mutation in the respective gene. Genes depicted were filtered for genes with established potential in lymphoma (see Supplemental Table 5). **B** Oncoplot representing recurrent copy number alterations detected in rrDLBCL tumor samples (*n* = 132). Predicted event is represented by color (Amplification [Amp], dark red; Gain, light red; Deletion [Del], dark blue; Loss, light blue). Genomic regions depicted were filtered for locations with established potential in lymphoma (see Supplemental Table 6). **C** Forest plot and enrichment analysis comparing recurrent non-silent somatic single variants or insertions/deletions between rrDLBCL (*n* = 204) and ndDLBCL (*n* = 382) tumors. OR > 1 indicates enrichment in rrDLBCL tumors. **D** Forest plot and enrichment analysis comparing recurrent copy number alterations between rrDLBCL (*n* = 132) and ndDLBCL (*n* = 365) tumors. OR > 1 indicates enrichment in rrDLBCL. **E** Summary visualizing frequency of recurrent non-silent somatic single variants or insertions/deletions and copy number alterations in rrDLBCL and ndDLBCL tumors. Alteration type represented by color (mutation, green; gain, red; loss, blue). For clarity, only SNV and CNV data from genes/regions from **C**, **D** are shown. Bubble size represents −Log10(*p* value). Points above dotted line indicate enrichment in ndDLBCL tumor samples, points below dotted line indicate enrichment in rrDLBCL tumor samples. **F** LymphGen classification in ndDLBCL (*n* = 349) and rrDLBCL (*n* = 131) tumor samples.

Single-nucleotide variants (SNVs) and short somatic insertions or deletions were called across rrDLBCL samples for a median tumor mutation burden (TMB) of 134.5 per patient (range 9–4534) and 2.69 per Mb (range 0.18–90.76). Evaluating SNVs and insertion/deletion events across a landscape of genes or gene regions with established potential in lymphoma (Supplemental Tables 5 and 6), mutations affecting *KMT2D*, *PCLO*, *IGLL5*, *TP53*, and *CREBBP* were most frequently observed in rrDLBCL tumors (Fig. 1A). Evaluating copy number variants (CNVs), rrDLBCL tumors were dominated by gains/amplifications in regions affecting 18q21.33 (*BCL2* locus), 19q13.42 (*BIRC8*), 1q32.1 (*MDM4*, *IL10*), 1q23.3 (*CD244*, *PBX1*), and 8q24.21 (*MYC*) and losses/deletions in regions affecting 17p11.2 (*NCOR1*), 17p13.1 (*TP53*, *EIF5A*), 6q23.3 (*TNFAIP3*, *IL20RA*, *IL22RA2*), 6q21 (*FOXO3*, *HDAC2*), and 1p36.32 (*TP73*; Fig. 1B).

### Genomic enrichments in rrDLBCL

With the intent to describe genomic variants in rrDLBCL and enrichment for events occurring more frequently in the diagnostic or relapsed setting, we next leveraged a catalog of ndDLBCL tumor samples recently described by our group [27]. We observed a reduction in total SNVs and insertion/deletions in rrDLBCL compared to ndDLBCL (134.5/pt, 2.69/Mb vs 181.0/pt, 3.62/Mb; *p* = 1.5e-5, effect size (*r*) = −0.18; Supplemental Fig. 4A). rrDLBCL TMB interrogated here is a modest reduction compared to literature reporting non-synonymous somatic mutation rate of 3.2/Mb and 3.3/Mb in smaller studies of both diagnostic and rrDLBCL [20, 23, 43].

To explore mutation signatures attributed to tumorigenic processes in rrDLBCL, we evaluated single base substitutions (SBS) in the context of signature patterns described in the COSMIC database [44, 45]. Mutational patterns in rrDLBCL displayed elevated weight toward SBS signatures related to defective DNA mismatch repair (SBS15, *p* = 0.003, *r* = 0.12; SBS6, *p* = 0.13, *r* = 0.06), activity of activation-induced cytidine deaminase (SBS84, *p* = 0.049, *r* = 0.08), and chemotherapy treatment (SBS86, *p* = 0.002, *r* = 0.13; Supplemental Fig. 4B).

Comparing mutation frequency between rrDLBCL and ndDLBCL, low-frequency mutations including *MS4A1*, *HIST2H2BE*, *CD79A*, *NAV1*, *PPP1R9B*, and *STAT6* were more frequently present in rrDLBCL (Fig. 1C, Supplemental Fig. 4C). Both truncating and missense mutations in the *MS4A1* gene (encoding the CD20 protein) were almost exclusively present in the CD20 transmembrane domain (Supplemental Fig. 4D), similar to previous reports in rrDLBCL [25]. Mutations affecting *TNFRSF14*, *S1PR2*, *GNA13*, and *EZH2* were among genes more frequently observed in ndDLBCL. Further, CNV events were exclusively observed at a higher frequency in rrDLBCL–highlighted by gains at 19q13.42 (*ZNF628*, *NAT14*, *SSC5D* locus), 8q24.21 (*CASC11*, *MYC*), 18q21.33 (*PIGN*, *BCL2*), 1q23.3 (*FCGR2B*), and 1q32.1 (*TMCC2*, *IL10*), and losses at 4q35.1 (*CYP4V2*, *TLR3*), 17p11.2 (*TOP3A*, *FOXO3B*), 19p13.3 (*CD70*), 2q22.2 (*KYNU*, *ARHGAP15*), and 17p13.1 (*TP53*; Fig. 1D). SNV and CNV frequency for enriched genes/loci between rrDLBCL and ndDLBCL samples are visualized in Fig. 1E. A higher proportion of LymphGen-subtype A53 were observed in rrDLBCL (38.9% vs 15.5%, *p* < 0.001), as were a modest reduction in the frequency of EZB (10.7% vs 15.8%, *p* = 0.19) and BN2 (2.3% vs 5.4%, *p* = 0.22; Fig. 1F). ‘Genetically Composite’ LymphGen cases appeared more abundant in rrDLBCL (9.9% vs. 4.3%; *p* = 0.03), while tumors in both rrDLBCL and ndDLBCL calculated >90% probability for EZB and A53 core subtypes.

### GCB and DZsig+ tumors highlight aggressive molecular features in rrDLBCL

**Fig. 2:**
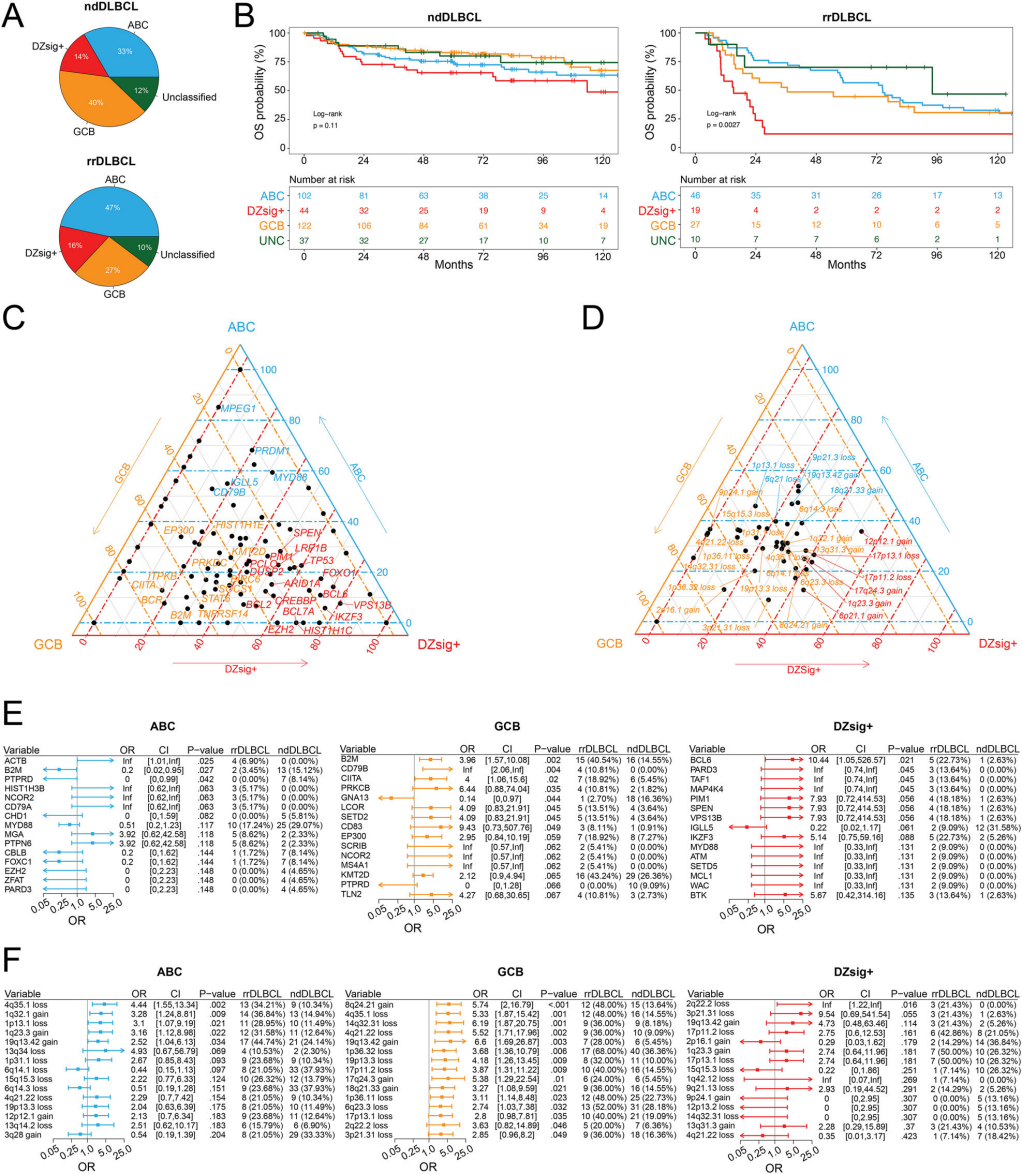
High-risk molecular features and poor outcomes in rrDLBCL tumors harboring dark-zone COO gene expression signature. **A** COO composition in ndDLBCL (ABC, *n* = 102; GCB, *n* = 122; DZsig+, *n* = 44; Unclassified, *n* = 37) and rrDLBCL (ABC, *n* = 68; GCB, *n* = 39; DZsig+, *n* = 24; unclassified, *n* = 15) tumors. **B** Kaplan–Meier estimation of overall survival (OS, from the time of original diagnosis) for patients from ndDLBCL cohort (left, *n* = 305) and rrDLBCL cohorts (right, *n* = 102) stratified by COO subtype. Patients analyzed at the indicated time points are shown in the table. ABC Blue, GCB yellow, Unclassified green, DZsig+ red. *P* value determined via Log-rank test. **C** Ternary plot visualizing the relative frequency of recurrent non-silent somatic single variants or insertions/deletions in rrDLBCL tumors stratified by COO subtypes ABC (*n* = 58, blue), GCB (*n* = 37, yellow), and DZsig+ (*n* = 22, red). Mutations affecting >15% of tumors within each respective subtype were labeled. **D** Ternary plot visualizing the relative frequency of recurrent copy number alterations in rrDLBCL tumors stratified by COO subtypes ABC (*n* = 37, blue), GCB (*n* = 25, yellow), and DZsig+ (*n* = 14, red). CNVs affecting >25% of tumors within each respective subtype were labeled. **E** Forest plot and enrichment analysis comparing recurrent non-silent somatic single variants or insertions/deletions between rrDLBCL and ndDLBCL tumors stratified by COO subtypes ABC (left; *n* = 58 rrDLBCL, *n* = 86 ndDLBCL), GCB (middle; *n* = 37 rrDLBCL, *n* = 110 ndDLBCL), and DZsig+ (right; *n* = 22 rrDLBCL, *n* = 38 ndDLBCL). OR > 1 indicates enrichment in rrDLBCL. **F** Forest plot and enrichment analysis comparing recurrent copy number alterations between rrDLBCL and ndDLBCL tumors stratified by COO subtypes ABC (left; *n* = 38 rrDLBCL, *n* = 87 ndDLBCL), GCB (middle; *n* = 25 rrDLBCL, *n* = 110 ndDLBCL), and DZsig+ (right; *n* = 14 rrDLBCL, *n* = 38 ndDLBCL). OR > 1 indicates enrichment in rrDLBCL.

Compared to ndDLBCL, rrDLBCL samples were enriched for the ABC subtype (47% vs 33%; *p* = 0.01), and fewer GCB-subtype tumors (27% vs 40%; *p* = 0.006, Fig. 2A). Among COO subtypes, there was no difference in the distribution of age at original diagnosis in cases from ndDLBCL and rrDLBCL cohorts, although rrDLBCL patients >80 years of age at diagnosis predominantly contained ABC subtype tumors (Supplemental Fig. 5A). rrDLBCL patients with DZsig+ subtype tumors experienced a shorter DTI during the diagnostic phase of their disease than patients with ABC (*p* = 0.001, *r* = −0.40) or GCB (*p* = 0.04, *r* = −0.32) tumors (Supplemental Fig. 5B). Amongst rrDLBCL patients, variance in overall survival (measured from time of original diagnosis; log-rank *p* = 0.003) was observed between COO subtypes, with poor outcomes in patients harboring DZsig+ (median 15.0 mo) and GCB (36.5 mo) compared to ABC tumors (74.4 mo; Fig. 2B).

In rrDLBCL, somatic mutations affecting genes with established potential in lymphoma were more frequently present in GCB and DZsig+ tumors relative to ABC tumors (Fig. 2C). Mutations affecting *IKZF3*, *BCL6*, *ARID1A*, *CREBBP*, *TP53*, and *SPEN* were more frequently present in DZsig+ tumors, and *KMT2D*, *B2M*, *SOCS1*, *CIITA*, *EP300*, and *STAT6* mutations were more frequently observed with in GCB rrDLBCL. rrDLBCL mutations appearing more frequently in ABC tumors included *MPEG1*, *PRDM1*, *MYD88*, *IGLL5*, and *CD79B*. Alternatively, nearly all CNV events in rrDLBCL were observed at a higher frequency in GCB tumors, headlined by gains at 2p16.1, 9p24.1, and 8q24.21, or losses at 1p36.32, 14q32.31, 1p36.11, 3p21.31, and 19p13.3 (Fig. 2D).

Directly comparing mutation frequency between rrDLBCL and ndDLBCL tumors within respective subtypes, enrichment for a few mutations was observed between ABC tumors at diagnosis or relapse, with only *B2M* and *PTPRD* mutations appearing more frequently in ndDLBCL and *ACTB* mutations more frequent at relapse (Fig. 2E). Alternatively, GCB-subtype tumors featured numerous mutations enriched or depleted at relapse, including *GNA13* mutations in ndDLBCL and *B2M*, *CD79B*, *CIITA*, *PRKCB*, *LCOR*, *SET2D*, and *CD83* mutations in rrDLBCL. rrDLBCL DZsig+ tumors demonstrated enrichment for mutations in *BCL6*, *PARD3*, *TAF1* and *MAP4K4*, whereas only *IGLL5* mutations appeared modestly enriched in ndDLBCL.

Similarly, enrichment for CNV events was restricted to COO subtypes in rrDLBCL. Gains at 19q13.42 appeared as a consistent feature in rrDLBCL regardless of COO (Fig. 2F). ABC rrDLBCL featured enrichment for losses at 4q35.1 and gains/losses affecting chromosome 1 (1q32.1 gain, 1p13.1 loss, 1q23.3 gain). Increased genomic complexity was overall observed in GCB rrDLBCL, where samples at relapse demonstrated enrichment for several CNV events, including gains at 8q24.21, 17q24.3, and 18q21.33, and losses at 4q35.1, 14q32.31, 4q21.22, and 1p36.32, among others. Enrichment for CNV events in DZsig+ rrDLBCL tumors was limited, only observing losses at 2q22.2 and 3p21.31. The LymphGen A53 classification was more abundantly observed in ABC (43% vs 17%, *p* = 0.005) and GCB (40% vs 14%, *p* = 0.009) rrDLBCL (Supplemental Fig. 5C).

### Transcriptional programming in rrDLBCL

**Fig. 3:**
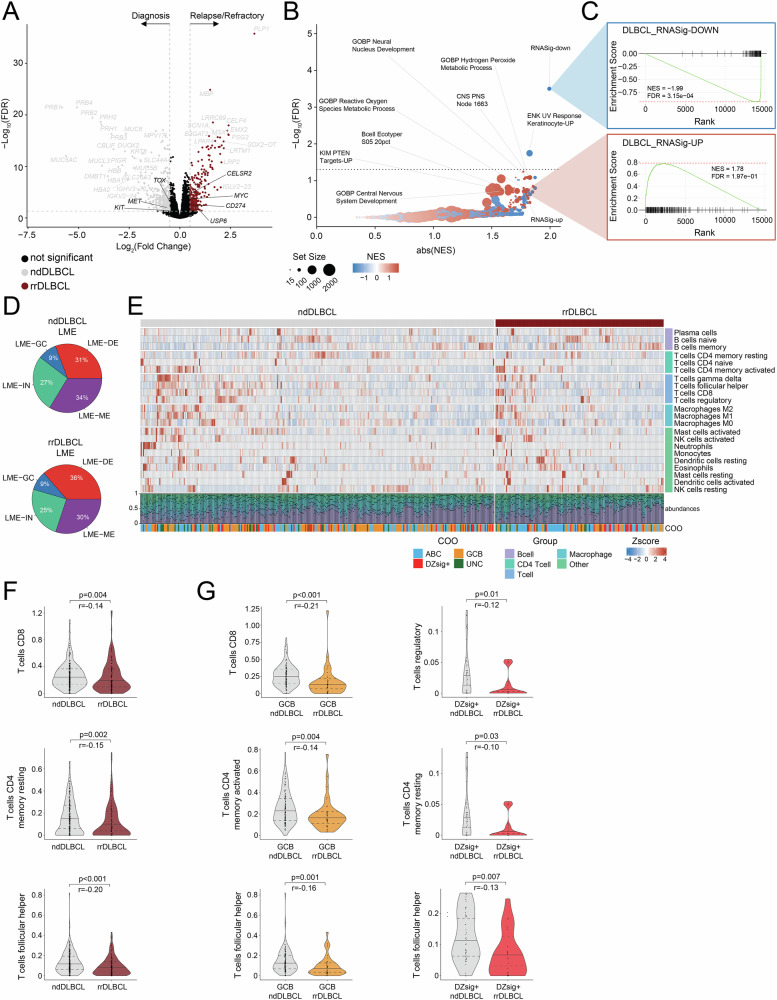
Transcriptional programming in rrDLBCL. **A** Differential gene expression analysis from bulk RNA-sequencing in rrDLBCL (*n* = 144) and ndDLBCL (*n* = 303) tumor samples. Genes with significant enrichment (FDR < 0.05, |Log2FC|>0.5) in rrDLBCL tumors (maroon) or ndDLBCL tumors (gray) are indicated by color. Dotted line indicates −Log10(FDR) = 0.05 and Log2|FoldChange| = 0.5. **B** Gene set enrichment analysis (GSEA) describing disrupted pathways as indicated from differential gene expression in **A**. *X* axis represents the absolute value of normalized enrichment score (NES), *y* axis represents −Log10(adj *p* value). Gene set size indicated by bubble size. NES indicated by color (Blue–negative enrichment, red–positive enrichment). Dotted line indicates −log_10_(0.05). **C** Leading edge plots visualizing enrichment score for DLBCL_RNASig-DOWN and DLBCL_RNASig-UP gene sets from GSEA in (**B**). **D** Classification of lymphoma microenvironment (LME) signatures inferred from gene expression profiling in ndDLBCL (*n* = 303) and rrDLBCL (*n* = 143) tumor samples. LME-DE red, LME-GC blue, LME-IN teal, LME-ME purple. **E** Cell type abundance of the associated lymphoma microenvironment inferred from gene expression analysis using the CIBERSORTx tool in ndDLBCL (*n* = 303) and rrDLBCL (*n* = 144) tumor samples. Cell types are grouped by rows. COO annotation is indicated below. **F** Enrichment of inferred T-cell subtypes from **E**. *p*-value determined using Wilcoxon rank sum test. **G** Enrichment of inferred T-cell subtypes among GCB (*n* = 39 rrDLBCL, *n* = 121 ndDLBCL) and DZsig+ (*n* = 22 rrDLBCL, *n* = 44 ndDLBCL) tumors from **E**. *p*-value determined using Wilcoxon rank sum test.

To evaluate the transcriptional profile of rrDLBCL, we conducted RNA sequencing in rrDLBCL tumors (*n* = 144) and compared against profiles established from ndDLBCL (*n* = 303), with any overlapping (paired ndDLBCL and rrDLBCL) cases removed from analysis. Differential expression analysis revealed minimal transcriptional variance between relapsed and diagnostic tumors, observing 709 up- or down-regulated genes (FDR < 0.05, |Log2FC | >0.5) including enrichment for genes with established potential in lymphoma in rrDLBCL such as *CELSR2* (Log2FC = 0.51, FDR = 1.95e-5), *MYC* (Log2FC = 0.55, FDR = 2.06e-4), *CD274* (*PD-L1*; Log2FC = 0.52, FDR = 3.19e-3), and *USP6* (Log2FC = 0.76, FDR = 4.25e-3; Fig. 3A). Transcriptional programming within COO subtypes was also similar between diagnostic and relapsed tumors, however overexpression genes including *CELSR2* (Log2FC = 0.53, FDR = 0.02), *MYBPC2* (Log2FC = 1.68, FDR = 4.77e-4), *MYC* (Log2FC = 0.61, FDR = 0.04), and *MYOM2* (Log2FC = 1.33, FDR = 6.93e-3) was observed in ABC rrDLBCL, and modest downregulation of *MS4A1* (Log2FC = −0.77, FDR = 0.33) in DZsig+ rrDLBCL (Supplemental Fig. 5D).

Our previous work in DLBCL utilized transcriptional programming to establish a high-risk signature genomic classification tool [27]. Here, we observed significant negative enrichment for genes whose reduced expression is associated with high risk for EFS24 failure (RNASig-DOWN; NES = −1.99, FDR = 3.15e-4) and modest positive enrichment for genes whose elevated expression is associated with high risk for EFS24 failure (RNASig-UP; NES = 1.78, FDR = 0.197; Fig. 3B, C). Furthermore, gene set enrichment analysis (GSEA) identified positive enrichment for genes contributing to the Ecotyper classification B-cell state S05 in rrDLBCL (NES = 1.66, FDR = 0.181; Fig. 3B).

RNA-sequencing was further utilized to estimate the diversity and composition of the lymphoma microenvironment (LME) [46]. Minimal changes in the distribution of LME classifications were observed between ndDLBCL and rrDLBCL, although an LME-depleted (LME-DE) signature was slightly more evident in rrDLBCL (36% vs 31%; *p* = 0.24, Fig. 3D). The CIBERSORTx tool [47] was employed to infer cell type abundance and corroborated LME classifications, observing a similar distribution and activity of immune cell infiltrates in ndDLBCL and rrDLBCL (Fig. 3E). Evidence of an LME-depleted class was further evident in rrDLBCL when evaluating individual cell types, including a reduction in abundance of CD8 + T cells (*p* = 0.004, *r* = −0.14), CD4 + T resting memory cells (*p* = 0.002, *r* = −0.15), and T follicular helper cells (*p* < 0.001, *r* = -0.20; Fig. 3F). LME signature abundance followed patterns of COO subtype with stronger LME involvement clustering in ABC/Unclassified tumor samples (Supplemental Fig. 5F). Within COO subtypes, an LME-depleted class was again evident in rrDLBCL including reduction of CD8 + T cells (*p* < 0.001, *r* = −0.21) CD4 + T activated memory cells (*p* = 0.004, r = −0.14), and T follicular helper cells (*p* = 0.001, *r* = −0.16) in GCB rrDLBCL and reduction of regulatory T cells (*p* = 0.01, r = −0.12,), CD4 + T resting memory cells (*p* = 0.03, *r* = −0.10), and T follicular helper cells (*p* = 0.007, *r* = −0.13) and in DZsig+ rrDLBCL (Fig. 3G).

### Primary refractory DLBCL and mechanisms of early relapse/refractory disease

**Fig. 4:**
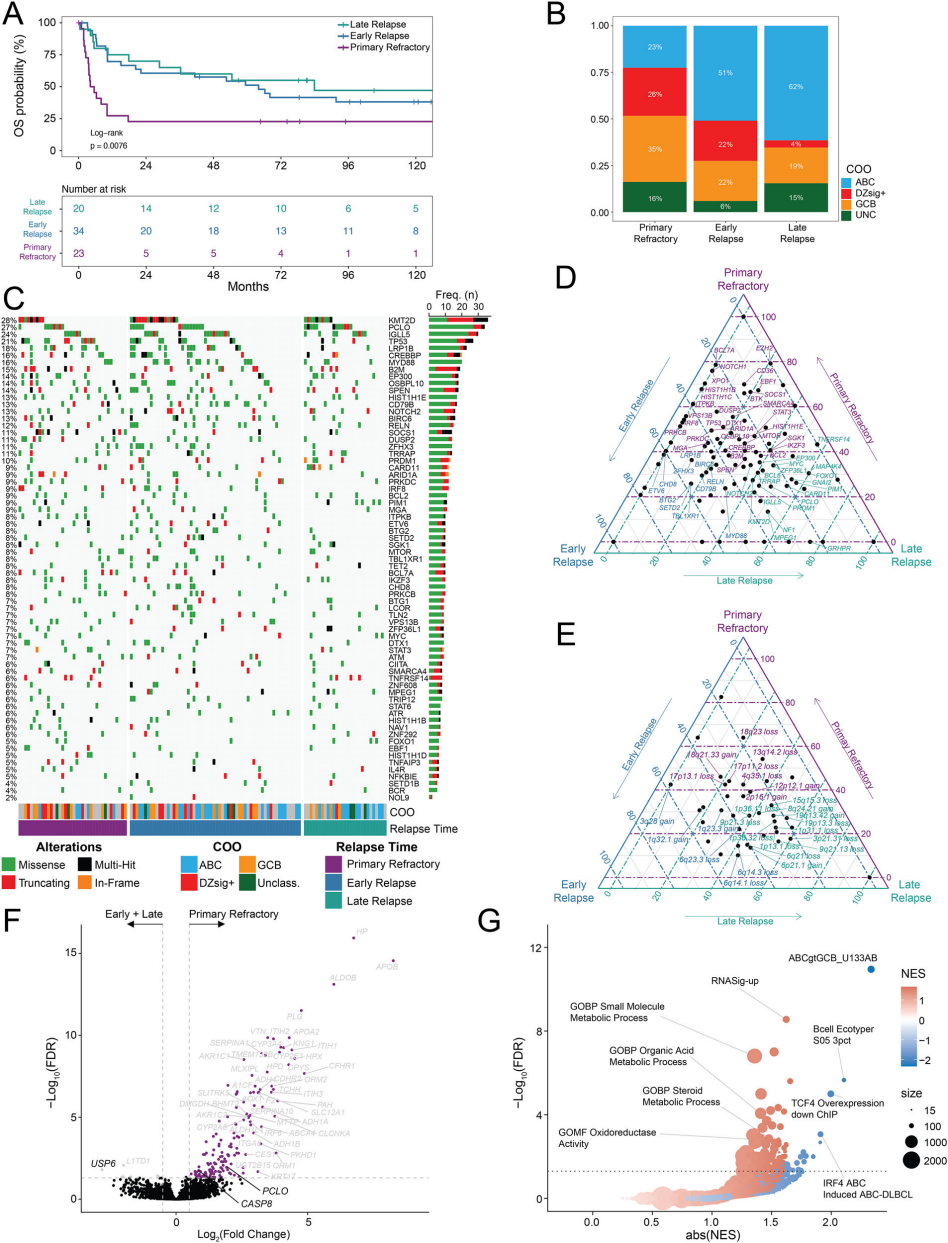
Relapse timing and tumor biology. **A** Kaplan–Meier estimation of overall survival (OS) for rrDLBCL patients stratified by the time at which their first relapse sample (R1) was collected. Patients available for analysis at the indicated time points are shown in the table. Primary refractory (*n* = 23)–Purple, early relapse (*n* = 34)–blue, late relapse (*n* = 20)–teal. *P* value determined via Log-rank test. **B** Proportion of COO among bins grouping rrDLBCL tumors by time at which the relapse sample was collected. Primary refractory, *n* = 31; early relapse, *n* = 51; late relapse, *n* = 26. ABC–blue, DZsig+–red, GCB–orange, Unclassified (UNC)–green. **C** Oncoplot representing recurrent non-silent somatic single variants or insertions/deletions detected in rrDLBCL tumor samples (*n* = 127) grouped by time at which the relapse sample was collected. Primary refractory, *n* = 38; early relapse, *n* = 60; late relapse, *n* = 29. Predicted functional consequence represented by color (missense, green; truncating, red; in-frame, orange; multi-hit, black). The bar plot (right) represents the sum of samples with a mutation in the respective gene. Genes depicted were filtered for genes with established potential in lymphoma (see Supplemental Table 5). **D** Ternary plot visualizing the relative frequency of recurrent non-silent somatic single variants or insertions/deletions in rrDLBCL tumors stratified by time at which the relapse sample was collected. Primary refractory (*n* = 38, purple), early relapse (*n* = 60, blue), late relapse (*n* = 29, teal). Mutations affecting >10% of tumors within each respective group were labeled. **E** Ternary plot visualizing the relative frequency of recurrent copy number alterations in rrDLBCL tumors stratified by time at which the relapse sample was collected. Primary refractory (*n* = 22, purple), early relapse (*n* = 26, blue), late relapse (*n* = 13, teal). Mutations affecting >25% of tumors within each respective group were labeled. **F** Differential gene expression analysis from bulk RNA-sequencing in rrDLBCL comparing primary refractory tumors (*n* = 30) against early + late relapse tumors (*n* = 77). Genes with significant enrichment (FDR < 0.05, |Log2FC|>0.5) are indicated by color. Genes with −Log(FDR) > 1.5 & |Log2FC|>2.5 are labeled in gray, while genes with established potential in lymphoma are labeled in black. **G** Gene set enrichment analysis (GSEA) describing disrupted pathways as indicated from differential gene expression between cases from **F**. X-axis represents absolute value of normalized enrichment score (NES), *y* axis represents −Log10(FDR). Dotted line indicates −Log_10_(0.05). Gene set size indicated by bubble size. NES indicated by color (Blue negative enrichment, red positive enrichment).

With regard to relapse timing, patient outcomes appeared consistent with previous reports [8, 48, 49] where rrDLBCL patients that progressed or relapsed less than 6 months from initial diagnosis (defined here as primary refractory disease, 30% of eligible rrDLBCL) demonstrated inferior OS (measured from the time the patient’s first relapse sample was collected–median 4.8 mo.) compared to those relapsing between 6 and 24 months (early relapse, 46% of eligible rrDLBCL;–median 64.0 mo.) or after 24 months (late relapse, 24% of eligible rrDLBCL; median–83.9 mo.; Log-rank *p* = 0.008; Fig. 4A). Patients with primary refractory disease featured a more even refined COO distribution (ABC–23%, GCB–35%, DZsig+–26%) than patients with early (ABC–51%, GCB–22%, DZsig+–22%) or late relapses (ABC–62%, GCB–19%, DZsig+–4%), which were predominantly ABC (Fig. 4B; Supplemental Table 7). Somatic mutation analysis in rrDLBCL grouped by relapse timing revealed that mutations in *TP53*, *SOCS1*, *CD36*, *BTK*, and *EZH2* were more frequently observed in primary refractory tumors (Fig. 4C, D). Mutations affecting *CD79B* and *MYD88* were more frequently observed in early relapse cases. For CNV events, gains at 18q21.33 and losses at 18q23, 13q14.2, 17p13.1, 17p11.2 and 4q35.1 were more frequently observed in primary refractory tumors (Fig. 4E). Differential gene expression analysis in primary refractory against early + late relapse tumors combined revealed upregulation of *PCLO* and *CASP8* and downregulation of *USP6* among 168 differentially expressed genes (Fig. 4F). Here, GSEA identified significant positive enrichment for the high-risk RNASig-UP gene set (NES = 1.62, FDR = 2.78e-9) among several metabolic processes and negative enrichment for the B-cell state S05 Ecotyper classification (NES = −2.11, FDR = 2.21e-6) with additional ABC subtype-specific gene sets (Fig. 4G).

### Evolutionary dynamics among rrDLBCL tumors

**Fig. 5:**
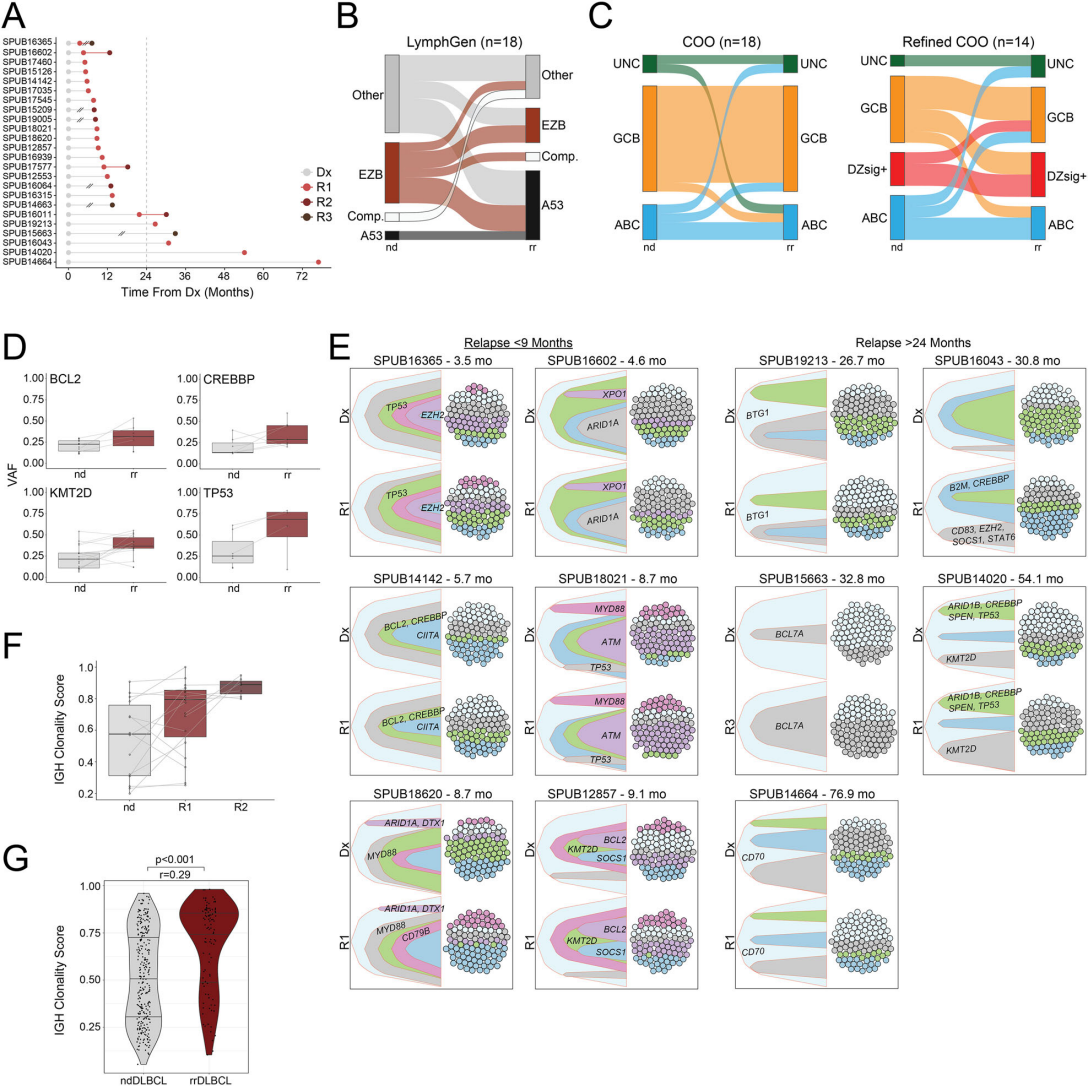
Patterns of clonal evolution in rrDLBCL tumors. **A** Time from diagnosis to relapse sample acquisition (months) from samples from **A**. Sample type indicated by color (Dx gray, R1 red, R2 dark red, R3 brown). Line breaks indicate a previous relapse event was clinically annotated, but no sample was available for analysis. The dashed line visualizes 24 months from diagnosis. **B** Sankey plots visualizing LymphGen (*n* = 18) molecular classifications in paired diagnostic and relapsed/refractory tumor samples. **C** Sankey plots visualizing COO (*n* = 18) and Refined COO (*n* = 14) molecular classifications in paired diagnostic and relapsed/refractory tumor samples. **D** Raw vaf assessed in *BCL2*, *CREBBP*, *KMT2D*, and *TP53* genes between paired diagnostic and relapsed/refractory tumor samples. Connecting lines indicate the same variant present in both paired samples from respective patients. **E** Clonal population structure in representative paired ndDLBCL and rrDLBCL samples inferred from PyClone-VI and ClonEvol tools using bulk SNV and CNV data as input, visualized by bell plot and sphere of cells. Colors represent distinctly inferred clones. Colored dots comprising spheres of cells are representative of the clonal abundance depicted in respective bell plots. Samples relapsing before 24 months are shown on the left, and samples relapsing after 24 months are shown on the right. Mutations in select lymphoma-driving genes are shown in affected clones. **F** IGH clonality score determined from the TRUST4 tool using bulk RNA-sequencing as input in paired ndDLBCL and rrDLBCL (R1 and R2) tumor samples. Dots represent individual samples. Lines connect paired samples. **G** IGH clonality score determined from the TRUST4 tool in ndDLBCL (*n* = 303) and rrDLBCL (*n* = 114) tumor samples. Dots represent individual samples. *P* value determined using the Wilcoxon rank sum test.

To investigate evolutionary dynamics in rrDLBCL, we next evaluated 24 patients with serial biopsies collected at diagnosis and relapse. Reflective of the cohort of rrDLBCL samples in this study, relapsed samples were collected prior to 24 months from diagnosis in 79% of paired cases (Fig. 5A). LymphGen phenotypes appeared to be fluid between paired samples with multiple ‘other’ or EZB diagnostic tumors called as A53 in the paired relapsed sample (Fig. 5B), while RNA-seq based genetic classifiers remained largely concordant between paired diagnostic and relapsed tumors (COO–Cohen’s kappa = 0.55; Refined COO–Cohen’s kappa = 0.4; Fig. 5C). The majority of shifts in Refined COO after relapse occurred between GCB and DZsig+ classifications. The variant allele frequency (VAF) of recurrently mutated genes (e.g., *BCL2*, *CREBBP*, *KMT2D*, *TP53*) was consistently elevated in paired rrDLBCL samples, suggesting these rrDLBCL tumors feature selection and expansion of a tumor clone that is present amongst the tumor burden at diagnosis (Fig. 5D). In accordance, clonal expansion of tumor populations harboring mutations in genes with established potential in lymphoma was observed in rrDLBCL tumors, noting expansion in clonal frequency of populations with *ATM*, *B2M*, *BCL2*, *CD79B*, *CREBBP*, *KMT2D*, *SOCS1*, *STAT6*, and *TP53* mutations (Fig. 5E). Clonal dynamics estimation revealed a linear pattern of stepwise evolution along a singular clonal ‘trunk’ predominantly evident in cases relapsing within 9 months, which was in stark contrast to patterns of branched clonal evolution more frequently observed in later relapses.

We further employed the TRUST4 [50] algorithm to reconstruct BCR repertoires from bulk RNA-sequencing. Evaluation of CDR3 sequence identity in paired samples revealed reduction in BCR repertoire diversity (inversely related to clonality score) in subsequent tumor samples, indicative of clonal enrichment at time of relapse and is supportive of an evolutionary dynamic driven by clonal selection and expansion (Fig. 5F). In paired samples, the most abundant CDR3aa usage in relapsed samples was identified as dominant usage or subclonal usage in the previous sample in all cases, indicating hypermutation of the IGH locus has continued after diagnosis in some cases. BCR repertoire analysis in all ndDLBCL and rrDLBCL samples further supports the observation of clonal selection and expansion, where ndDLBCL tumors were heterogeneously comprised of diverse, polyclonal BCR repertoire and rrDLBCL tumors were more consistently comprised of a highly clonal tumor population with minimal diversity in BCR repertoire (*p* < 0.001, *r* = 0.29; Fig. 5G).

## Discussion

This study nearly doubles the collective abundance of high-throughput sequencing in rrDLBCL presented to date, and with robust clinical annotations, we provide detailed characterization of this high-risk patient population. Our study is supportive of the hypothesis that many diagnostic DLBCL tumors contain genomic signatures that converge on a phenotype of innate chemoresistance and are predisposed to a trajectory of relapsed/refractory clinical events. Collectively, we find that rrDLBCL tumors are prone to DNA losses, gains, and rearrangements that result in amplification of lymphoma driver genes (e.g., *MYC*, *BCL2*, *IL10*) or depletion of critical tumor suppressor genes (e.g., *TOP3A*, *CD70*, *TP53*). The mutational landscape of rrDLBCL tumors remains largely similar to ndDLBCL, although enrichment for low frequency mutations such as *MS4A1* (3.4% vs 0.5%), *CD79A* (3.9% vs 1.0%), or *STAT6* (7.8% vs 3.7%) suggest mechanisms of resistance to anti-CD20 driven therapy, constitutive activation of B-cell receptor signaling, and remodeling of the tumor microenvironment are highly specific features in a subset of DLBCL tumors refractory to immunochemotherapy [24, 25, 51, 52].

Enrichment for *MS4A1* mutations (encoding the CD20 protein) at low frequency (<5% of rrDLBCL cases) suggests this mechanism, likely driving progression via rituximab resistance, should not be considered a major factor for the majority of rrDLBCL tumors. None of the rrDLBCL *MS4A1* missense mutations were predicted to directly affect residues comprising the rituximab epitope [53], instead likely resulting in impaired and unstable protein folding [25]. CD20 downregulation may be a more consistent mechanism in DZsig+ rrDLBCL tumors, as seen by RNA-seq in Supplemental Fig. 5D, yet understanding mechanisms facilitating CD20 downregulation via alternative mechanisms or post-translational modifications [54] in the context of DZsig+ tumors remains to be explored. Alternatively, microenvironment reprogramming mechanisms from *TNFRSF14* (6.9% vs 15.2%), *S1PR2* (1.0% vs 5.8%), *GNA13* (4.9% vs 11.8%), or *EZH2* (4.9% vs 11.5%) mutations enriched in ndDLBCL (of which, the later three are notably absent in ABC subtype DLBCL) [55–59] may confer more sensitivity to standard immunochemotherapy.

While numerous large-scale sequencing studies have produced a comprehensive database for evaluating the genomic complexity of ndDLBCL, few studies of limited size have addressed mechanisms supporting therapeutic resistance and relapse. Previously, Morin and colleagues [24] (*n* = 38 rrDLBCL) and Rushton and colleagues [25] (*n* = 135 rrDLBCL) utilized high-throughput sequencing techniques to evaluate genetic variants with higher frequency at relapse and for evidence of clonal selection. It is encouraging that the cohort of rrDLBCL samples presented here is congruent with several of these previously established results, reiterating the notion that the landscape of genetic variants in rrDLBCL is highly concordant with what is observed at diagnosis and that very few mutations demonstrate significant enrichment post-exposure to current frontline treatment.

Our cohort size additionally permitted enrichment analysis for genetic variants by refined COO subtype, and it is our hope that the results presented herein may contribute rationale for utilizing tumor mutation/CNV calling with genomic classifications to guide individualized patient risk stratification and influence recommendations for clinical management. We report that patients harboring rrDLBCL DZsig+ subtype tumors have truly dreadful prognosis, and while patients harboring GCB (non-DZsig+) subtype tumors have historically positive treatment outcomes, our study reports that patients with GCB tumors that do experience a relapse/refractory event have very poor survival even compared to patients with ABC and Unclassified subtype tumors. This implies distinct mechanisms are likely driving more advanced tumors within these subsets, and prospective identification of tumors with or without defined driving mechanisms could provide critical insight into the clinical trajectory of the affected patient.

Here, DZsig+ and GCB-subtype rrDLBCL tumors demonstrated distinct mutational profiles, observing several mutations in genes with established potential in lymphoma, predominantly impacting DZsig+ (*ARID1A*, *BCL2*, *BCL6*, *CREBBP*, *EZH2*, *IKZF3*, *SPEN*, *TP53*) and GCB (*CIITA*, *EP300*, *KMT2D*, *SOCS1*, *STAT6*) samples, and CNV events were observed with higher relative frequency in GCB rrDLBCL. Considering enrichment for mutations within rrDLBCL COO subtypes against diagnostic samples, recurrent mutations affecting genes including *CIITA*, *EP300*, *NCOR2*, *BCL6*, and *SPEN* in GCB and DZsig+ rrDLBCL tumors suggest disruption to these transcriptional/epigenetic regulatory molecules has a significant impact on downstream pathogenic mechanisms that contribute to LME reprogramming/immune evasion signatures in rrDLBCL and in partnership with COO subtyping should be considered high-risk variants. Further, it is of considerable interest that numerous genomic alterations appear enriched in rrDLBCL DZsig+ or rrDLBCL GCB tumors that have been previously described as defining features of ABC or Unclassified COO subtype diagnostic DLBCL [33, 60]. This includes enrichment for *BCL6* or *SPEN* mutations and mild enrichment for copy number events such as 13q31.3 gain (*MIRHG1* locus–mechanism of MYC activation) and 9p21 loss (*INK4*/*ARF* locus–loss of tumor suppressors *CDKN2A* and *CDKN2B*) in rrDLBCL DZsig+ tumors, or enrichment for *CD79B* mutations and copy number events such as 19q13.4 gain (*SPIB* locus–mechanism of BCR signaling) and 6q23.3 loss (*TNFAIP3* [A20] locus–mechanism of NF-κB activation) in rrDLBCL GCB tumors. Future studies should emphasize the evaluation of these genomic enrichments among COO subtypes to better identify aggressive cases.

The dynamics of DLBCL tumor evolution have been a topic of considerable interest [61], and Hilton and colleagues [8] (*n* = 221 rrDLBCL) recently reported on evolutionary dynamics in rrDLBCL, reporting notable differences in underlying biology and outcomes in patients with late relapse (>24 months). While our study does not match the extensive analysis of 129 serial DLBCL biopsies by Hilton et al., we provide invaluable data that similarly conclude distinct evolutionary mechanisms drive primary treatment resistance and early relapse. In our study, given that the median DTI in patients from the rrDLBCL cohort during the diagnostic phase of their disease was 15.0 days, we defined primary refractory disease as those with EFS failure prior to 6 months from initial diagnosis to solely capture patients with progression during or by the end of frontline treatment. These patients featured drastically poor outcomes and were heavily skewed against ABC subtype tumors in favor of GCB and DZsig+, the former being much more prevalent in early and late relapse cases. Lack of chemosensitivity and high rate of clinical progression highlight the urgent need for a robust understanding of disease-driving mechanisms in these cases. As such, further evaluation of mechanisms derived from *TP53* [62], *SOCS1* [63], *CD36* [64], *BTK* [65], or *EZH2* [66] mutations in primary refractory DLBCL may shed light on disease driving mechanisms in these highly aggressive cases and present opportunities for more targeted second line therapy for those not eligible for ACST or CAR-T. Given the differential distribution of refined COO subtypes between primary refractory vs early or late relapse DLBCL, however, further evaluation is necessary to address whether enrichment of genomic features is more strongly attributed to COO or relapse timing.

Clonal evolution analysis suggests the mutational landscape and transcriptional signatures driving rrDLBCL tumors are strongly present at diagnosis, especially in primary refractory cases, where, upon exposure to treatment, rrDLBCL tumors are comprised of a highly clonal population of B cells enriched for lymphoma-driving genomic variants supporting therapeutic resistance. Evaluating patterns of clonal evolution in diagnostic tumor samples may be a useful tool for predicting cases likely to progress with relapse, where, as shown in paired analysis here, tumor evolution along a linear pattern with minimal clonal branching was highly evident in primary refractory and early relapse cases. With the data presented in this study, in late relapse cases, while the patient experiences an initial response to frontline treatment, selective pressure and continued hypermutation likely lead to outgrowth of branched tumor clones. From these observations, we may conclude that routine serial tumor sampling with extensive genomic analyses is not then needed in primary treatment-resistant cases, as the genomic profile of the relapsed/refractory tumor is likely to be highly similar to what has been analyzed in the diagnostic sample. Alternatively, non-invasive techniques (mutation analysis of ctDNA from liquid biopsies) to evaluate mechanisms driving later relapse tumors (>6 months) should be considered, as significant divergence from the original diagnostic sample is likely.

Collectively, we present an update to the genomic landscape of rrDLBCL tumors with optimism that a more detailed understanding of mechanisms driving relapsed/refractory disease may guide targeted therapeutic strategies for the management of rrDLBCL and strongly encourage the adoption of comprehensive genomic characterization of DLBCL tumors as routine clinical practice. As financial barriers and turnaround time for high-throughput sequencing efforts continues to decline, the approach utilized in this study to determine COO subtypes from gene expression analysis paired with the potential for a targeted sequencing panel of *n* = 292 genes and copy number evaluation of *n* = 45 genomic locations may sufficiently provide clinicians and investigators information to identify high-risk patient populations and proactively strategize therapeutic management beyond standard immunochemotherapy.

For example, patients harboring GCB-subtype rrDLBCL tumors may be strong candidates for intervention with a lenalidomide-based/inspired regimen (e.g., Lenalidomide-rituximab [R2], Tafasitamab-Lenalidomide [Tafa-Len], Golcadomide), as lenalidomide has been shown to downregulate expression of *PD-L1* in addition to downregulation of *MYC* and MYC target genes [67, 68]. Likewise, in line with the recently reported high-risk DLBCL cluster ‘A7’ featuring primarily ABC subtype tumors with abundant MYC pathway activation [69], enrichment for gains at 18q21 fostering upregulation of *TCF4*, a transcription factor known to regulate *MYC* expression [70], may support rrDLBCL patients as strong candidates for MYC pathway targeting agents (BETi). Further, elevated frequency of copy number gains at 2p16.1 (genomic region containing the *XPO1* gene) among GCB-subtype rrDLBCL tumors indicates these affected patients may be candidates for intervention with a small molecule XPO1 inhibitor [71, 72]. Or, due to enhanced frequency of mutations and/or copy number gains affecting *BCL2* in DZsig+ and GCB-subtype rrDLBCL tumors, a venetoclax-based therapeutic intervention may be considered [73]. With elevated frequency of *MS4A1* mutations and a decrease in *MS4A1* expression in DZsig+ rrDLBCL, CD19-directed CAR-T therapy may be a highly effective alternative strategy in patients no longer presenting tumors with surface expression of CD20. Targeted therapeutic strategies should be considered beyond these established molecules, as the development of a more personalized therapeutic approach remains an unmet need of critical importance in this high-risk patient population.

## Supplementary information

Supplemental Methods and Figures

Supplemental Tables

## Data Availability

DNA and RNA-sequencing data generated from study participants providing consent for release of genomic data have been deposited in dbGAP under accession code phs003868.v1.p1. Genomic data from the ndDLBCL patients utilized in this study can be accessed in dbGAP under accession code phs003634v1. ndDLBCL and rrDLBCL cohorts were compared to three publicly available ndDLBCL validation cohorts: NCI (*n* = 489; phs001444.v2.p1) [33], BCCA (*n* = 153) [21], and MSK-IMPACT (*n* = 220 [39]; Supplemental Fig. 2F). Additional data are available upon request.
