# Supplementary material for: Integrated genomics with refined cell-of-origin subtyping distinguishes subtype-specific mechanisms of treatment resistance and relapse in diffuse large B-cell lymphoma
Source: Blood Cancer J. 2025 Jul 12;15(1):120. doi: 10.1038/s41408-025-01326-5 (PMC12255755; doi:10.1038/s41408-025-01326-5)
Supplement: Supplementary file 1 — Supplemental Methods and Figures [file 41408_2025_1326_MOESM1_ESM.docx]

**DATA SUPPLEMENT**

**Integrated genomics with refined cell-of-origin subtyping distinguishes subtype-specific mechanisms of treatment resistance and relapse in diffuse large B cell lymphoma**

**Supplemental Methods**

**Study Populations**

Inclusion criteria for this study included: initial diagnosis of DLBCL (2016 WHO criteria), DLBCL with secondary histology of FL, EBV+, HGBCL with MYC and BCL2 and/or BCL6 rearrangement, HGBCL not otherwise specified. Exclusion criteria included: insufficient tumor (<50%), insufficient tissue in block, decalcified specimen, pathology review not consistent with DLBCL, T cell/histiocyte-rich large B cell lymphoma, or Primary mediastinal B cell lymphoma. Patients with a known history of indolent lymphoma with transformation to DLBCL were not included. Overall Survival (OS) was defined as time from initial diagnosis until death as a result of any cause. Event-free survival (EFS) was defined as time from diagnosis until relapse or progression, unplanned re-treatment of lymphoma after initial immunochemotherapy, or death as a result of any cause. Bulky disease was defined as >10cm mass by longest reported measurement in any direction (not including splenic lesions). B symptoms were defined as presentation with any one of: unexplained fever (>100.4°F/38°C), night sweats (soaking bed sheets, night/day sweats or drenching bed clothes) in the month prior to diagnosis, or unexplained weight loss (>10% of body weight) reported in the 6 months prior to diagnosis. LDH group ‘elevated’ was defined as LDH (U/L) > upper limit of normal (ULN). Primary cause of death (COD) was defined as ‘disease’ due to progressive lymphoma, ‘therapy’ due to therapy-related infection, cardiac, or other, or ‘other’ due to other causes not defined in ‘disease’ or ‘therapy’. Kaplan-Meier curves were used to display survival curves.

rrDLBCL and ndDLBCL patient characteristics were similar across age, number of extranodal sites, bulky disease, bone marrow involvement, and presence of B symptoms (**Supplemental Table 2**). The median duration from relapse/refractory tumor sample collection to time of last follow-up was 9.6 months for patients who did not meet EFS24 and 39.8 months in patients who successfully met EFS24 during the diagnostic phase of their disease (**Supplemental Figure 2A-B**). Most patients received frontline immunochemotherapy, primarily R-CHOP-based regimens, while subsequent treatment strategies in the rr setting were highly diverse (**Supplemental** **Table 6**). A majority of tumor samples were obtained as the patient’s first relapse event (R1, 60.8%), however later relapse samples (R2 [22.8%] - R10 [0.6%]) were collected (**Table 1**). Unless otherwise specified, high-throughput sequencing in rrDLBCL tumors was conducted on the first available relapse sample for each patient (**Supplemental Figure 2C**). Frequency of mutations from n=292 genes with established potential in DLBCL in ndDLBCL and rrDLBCL cohorts were compared to three ndDLBCL validation cohorts: NCI (n= 489)(1), BCCA (n=153)(2), and MSK-IMPACT (n=220 (3); **Supplemental Figure 2F**).

**DNA/RNA Sequencing and Analysis**

Methods for somatic mutation detection from WES and WGS in rrDLBCL samples were in accordance with methods outlined in Wenzl et al 2024(4) for ndDLBCL samples. Briefly, GATK best practices workflow was followed using Sentieon (v201808.05) implementations of picard and BWA. FASTQ files were trimmed using Cutadapt (v2.4) to remove sequencing adaptors and aligned to human reference build 38 using BWA-mem (0.7.17).(5) Duplicate reads were removed using Picard (v2.18). MarkDuplicates and base recalibration of alignments was performed using Base Quality Score Recalibration according to Genome Analysis Toolkit Best Practices. Single-nucleotide somatic variants (SNVs) and INDELs were called using Mutect2 from the GATK suite (v4.0.12) according to Genome Analysis Toolkit Best Practices and annotated by SnpEff(6) (v4.3i) using dbNSFP(7) 3.5c. For post alignment and somatic mutation calling, common variants which with a frequency higher than 10% percent in ExAC or gnomAD were removed. Mutations included required a depth of at least 10 in both tumor and normal, greater than 5% allele frequency in the tumor, less than 5% in the normal, and a minimum alternate allele depth of 3. All ndDLBCL tumor samples analyzed by WES were accompanied by matched constitutional (normal) DNA. For mutation calling in rrDLBCL tumor samples without matched normal DNA, the Mutect2 tumor-only pipeline was applied using the set of ‘normal’ mutations from gnomAD and dbSNP.

For LymphGen classification, data were prepared and submitted for classification as instructed by the online tool (<https://llmpp.nih.gov/lymphgen/index.php>) using only protein coding regions and excluding splice sites.(8) Frequency of mutations in genes relevant for LymphGen classification (n=125)(9) were highly concordant between ndDLBCL and rrDLBCL samples in our study and previously published studies on DLBCL(1, 2, 3) (**Supplemental Figure 2F**). Analysis of copy number variants (CNV) was carried out using Sclust(10) v1.1 using human genome reference build 38 (GRCh38) to align Bam files and annotated vcf files with default parameters. CNV analysis was conducted on n=365 ndDLBCL and n=132 rrDLBCL cases, all with matched constitutional DNA sequencing, in the same manner using sClust between both ndDLBCL and rrDLBCL. High concordance in CNV frequency was observed in rrDLBCL samples sequenced by WES (n=62) and WGS (n=70) (Pearson’s r = 0.82, CI = [0.68 – 0.90], p<0.001; **Supplemental Figure 2G**). Mutation signatures attributing to tumorigenic processes were evaluated by single base substitutions (SBS) in the context of signature patterns described in the COSMIC database(11). Weights of each mutational signature contributing to individual tumor samples were determined using deconstructSigs(12). For assessment of clonal evolution dynamics, SNV and CNA data was input to PyClone-VI(13) to deconvolve bulk sequencing data from paired ndDLBCL and rrDLBCL tumor samples and infer clonal population structure, and was then input to ClonEvol(14) for visualization of clonal ordering and evolution.

RNA fastq files were aligned to the human genome reference build 38 (GRCh38) using the Star aligner method.(15) Quantification of the aligned RNA sequencing data was carried out using salmon.(16) Differential expression was carried out using count data using the edgeR package.(17). EdgeR's genewise negative binomial generalized linear model was used to calculate genewise fold change and significance values. Protein coding genes were ranked using the metric -log10(pval)*log2(FoldChange). For Gene set enrichment analysis (GSEA), pathways C2, GO:BP, GO:MF, GO:CC, and Hallmark were queried from the Human MSigDB Collections(18). Genesets including RNA signatures DLBCL_EFS24_RNASig-UP, DLBCL_EFS24_RNASig-DOWN(4), and Lymphoma Signature Database (<https://lymphochip.nih.gov/signaturedb/>) were included. GSEA was performed on the ranked gene list and pathways using R package fgsea.(19)

Cell of origin (COO) in ndDLBCL samples was obtained from Wenzl et al(4), determined by the Lymph2Cx assay (NanoString; n=224)(20) or from RNA-Seq data (n=81) using the method described by Reddy et al(21) in diagnostic tumor samples. For rrDLBCL samples, COO status was assigned from RNA-Seq data in relapsed tumor samples according to the Reddy method and was shown to have excellent concordance (Pearson’s r = 0.95, CI=[0.93 – 0.96], p<0.001) with the gneSeqCOO method(22) (**Supplemental Figure 3E**). “Refined COO” subtype was assigned by applying the DHIT/DZsig method.(23, 24) The DHIT/DZsig method was adapted to the normalized RNAseq space by using published gene-level DHITsig coefficients to compute a linear DHITsig score. A threshold for this score was selected in the MER data as the highest threshold that captured all cases known to be double-hit by FISH, which yielded a classifier matching the expected prevalence of DHITsig+ cases. This signature was further extended to represent the DZsig classification by adding a third “indeterminate” group with approximately 10% prevalence between the positive and negative groups. Gene expression subgroups were assigned hierarchically, with COO taking precedence over DHITsig status for ABC tumors. GCB and UNC tumors that were DHITsig-pos were assigned to the DZsig+ group, whereas DHITsig-ind and DHITsig-neg tumors were assigned to their respective COO subgroups(24).

Log2(TPM+1) gene expression values for all cases were simultaneously submitted for Lymphoma Microenvironment (LME) deconvolution to the CIBERSORTx online tool.(25) Differences in absolute cell type abundance among groups were determined using the Wilcoxon rank sum test. The LM22 signature matrix was used and the data were permuted 500 times. The final data were reported as absolute abundances for each cell type. Gene expression was additionally analyzed by the Lymphoma Microenvironment tool (<https://github.com/BostonGene/LME>).(26) To process immunome data from RNA sequences into quantitated clonotypes we applied TRUST4 v v1.0.13-r474(27). Single clonotypes were identified as those with unique CDR3 nucleotide sequences. For B cells, the tumor clone bubbles in Figure 4F were colored similarly if they had an identical CDR3 nucleotide sequence with different VDJ combination. The biggest clone group was assigned as the tumor clone in each sample, unless the most abundant clone in the analyzed relapsed/refractory sample was present as a subclone in the diagnostic sample. Reported p-values were corrected for multiple testing using the Benjamini-Hochberg procedure (FDR).

**References**

1. Schmitz R, Wright GW, Huang DW, Johnson CA, Phelan JD, Wang JQ, et al. Genetics and Pathogenesis of Diffuse Large B-Cell Lymphoma. N Engl J Med. 2018;378(15):1396-407.

2. Arthur SE, Jiang A, Grande BM, Alcaide M, Cojocaru R, Rushton CK, et al. Genome-wide discovery of somatic regulatory variants in diffuse large B-cell lymphoma. Nat Commun. 2018;9(1):4001.

3. Zehir A, Benayed R, Shah RH, Syed A, Middha S, Kim HR, et al. Mutational landscape of metastatic cancer revealed from prospective clinical sequencing of 10,000 patients. Nat Med. 2017;23(6):703-13.

4. Wenzl K, Stokes ME, Novak JP, Bock AM, Khan S, Hopper MA, et al. Multiomic analysis identifies a high-risk signature that predicts early clinical failure in DLBCL. Blood Cancer J. 2024;14(1):100.

5. Li H, Durbin R. Fast and accurate short read alignment with Burrows-Wheeler transform. Bioinformatics. 2009;25(14):1754-60.

6. Cingolani P, Platts A, Wang le L, Coon M, Nguyen T, Wang L, et al. A program for annotating and predicting the effects of single nucleotide polymorphisms, SnpEff: SNPs in the genome of Drosophila melanogaster strain w1118; iso-2; iso-3. Fly (Austin). 2012;6(2):80-92.

7. Liu X, Wu C, Li C, Boerwinkle E. dbNSFP v3.0: A One-Stop Database of Functional Predictions and Annotations for Human Nonsynonymous and Splice-Site SNVs. Hum Mutat. 2016;37(3):235-41.

8. Wright GW, Huang DW, Phelan JD, Coulibaly ZA, Roulland S, Young RM, et al. A Probabilistic Classification Tool for Genetic Subtypes of Diffuse Large B Cell Lymphoma with Therapeutic Implications. Cancer Cell. 2020;37(4):551-68 e14.

9. Hilton LK, Ngu HS, Collinge B, Dreval K, Ben-Neriah S, Rushton CK, et al. Relapse Timing Is Associated With Distinct Evolutionary Dynamics in Diffuse Large B-Cell Lymphoma. J Clin Oncol. 2023:JCO2300570.

10. Cun Y, Yang TP, Achter V, Lang U, Peifer M. Copy-number analysis and inference of subclonal populations in cancer genomes using Sclust. Nat Protoc. 2018;13(6):1488-501.

11. Sondka Z, Dhir NB, Carvalho-Silva D, Jupe S, Madhumita, McLaren K, et al. COSMIC: a curated database of somatic variants and clinical data for cancer. Nucleic Acids Res. 2024;52(D1):D1210-D7.

12. Rosenthal R, McGranahan N, Herrero J, Taylor BS, Swanton C. DeconstructSigs: delineating mutational processes in single tumors distinguishes DNA repair deficiencies and patterns of carcinoma evolution. Genome Biol. 2016;17:31.

13. Gillis S, Roth A. PyClone-VI: scalable inference of clonal population structures using whole genome data. BMC Bioinformatics. 2020;21(1):571.

14. Dang HX, White BS, Foltz SM, Miller CA, Luo J, Fields RC, et al. ClonEvol: clonal ordering and visualization in cancer sequencing. Ann Oncol. 2017;28(12):3076-82.

15. Dobin A, Davis CA, Schlesinger F, Drenkow J, Zaleski C, Jha S, et al. STAR: ultrafast universal RNA-seq aligner. Bioinformatics. 2013;29(1):15-21.

16. Patro R, Duggal G, Love MI, Irizarry RA, Kingsford C. Salmon provides fast and bias-aware quantification of transcript expression. Nat Methods. 2017;14(4):417-9.

17. Robinson MD, McCarthy DJ, Smyth GK. edgeR: a Bioconductor package for differential expression analysis of digital gene expression data. Bioinformatics. 2010;26(1):139-40.

18. Liberzon A, Birger C, Thorvaldsdottir H, Ghandi M, Mesirov JP, Tamayo P. The Molecular Signatures Database (MSigDB) hallmark gene set collection. Cell Syst. 2015;1(6):417-25.

19. Korotkevich G, Sukhov V, Budin N, Shpak B, Artyomov MN, Sergushichev A. Fast gene set enrichment analysis. bioRxiv. 2021:060012.

20. Scott DW, Wright GW, Williams PM, Lih CJ, Walsh W, Jaffe ES, et al. Determining cell-of-origin subtypes of diffuse large B-cell lymphoma using gene expression in formalin-fixed paraffin-embedded tissue. Blood. 2014;123(8):1214-7.

21. Reddy A, Zhang J, Davis NS, Moffitt AB, Love CL, Waldrop A, et al. Genetic and Functional Drivers of Diffuse Large B Cell Lymphoma. Cell. 2017;171(2):481-94 e15.

22. Harris W, Cao Y, Morschhauser F, Salles G, Jiang Y, Bottos A, et al. gneSeqCOO: a novel method for classifying diffuse large B-cell lymphoma cell of origin based on bulk tumor RNA sequencing profiles. Leuk Lymphoma. 2025:1-8.

23. Ennishi D, Jiang A, Boyle M, Collinge B, Grande BM, Ben-Neriah S, et al. Double-Hit Gene Expression Signature Defines a Distinct Subgroup of Germinal Center B-Cell-Like Diffuse Large B-Cell Lymphoma. J Clin Oncol. 2019;37(3):190-201.

24. Alduaij W, Collinge B, Ben-Neriah S, Jiang A, Hilton LK, Boyle M, et al. Molecular determinants of clinical outcomes in a real-world diffuse large B-cell lymphoma population. Blood. 2023;141(20):2493-507.

25. Newman AM, Steen CB, Liu CL, Gentles AJ, Chaudhuri AA, Scherer F, et al. Determining cell type abundance and expression from bulk tissues with digital cytometry. Nat Biotechnol. 2019;37(7):773-82.

26. Kotlov N, Bagaev A, Revuelta MV, Phillip JM, Cacciapuoti MT, Antysheva Z, et al. Clinical and Biological Subtypes of B-cell Lymphoma Revealed by Microenvironmental Signatures. Cancer Discov. 2021;11(6):1468-89.

27. Song L, Cohen D, Ouyang Z, Cao Y, Hu X, Liu XS. TRUST4: immune repertoire reconstruction from bulk and single-cell RNA-seq data. Nat Methods. 2021;18(6):627-30.

**Supplemental Figures**

**Figure S1**

**
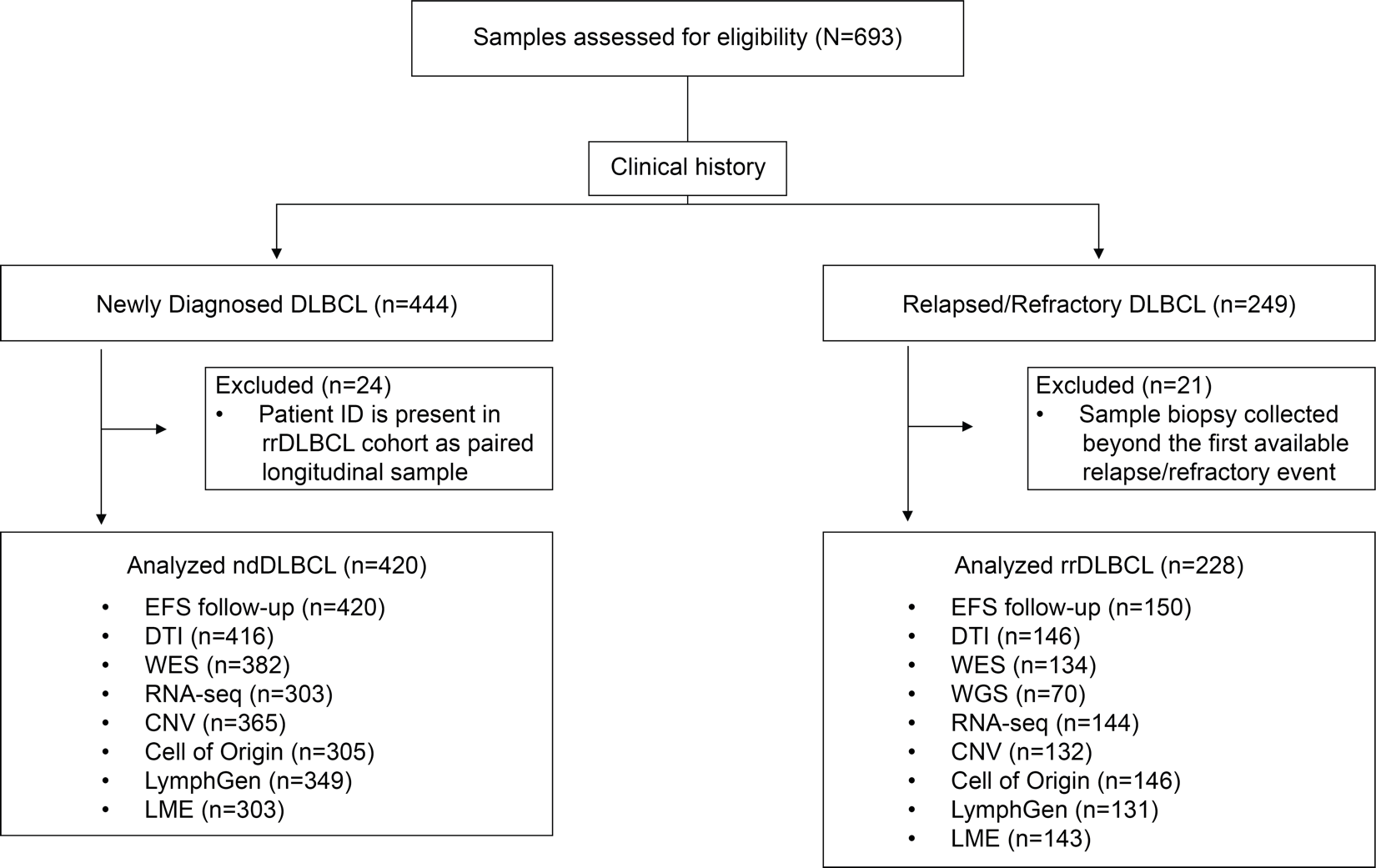
**

**Figure S1:** Consort diagram.

**Figure S2**

**
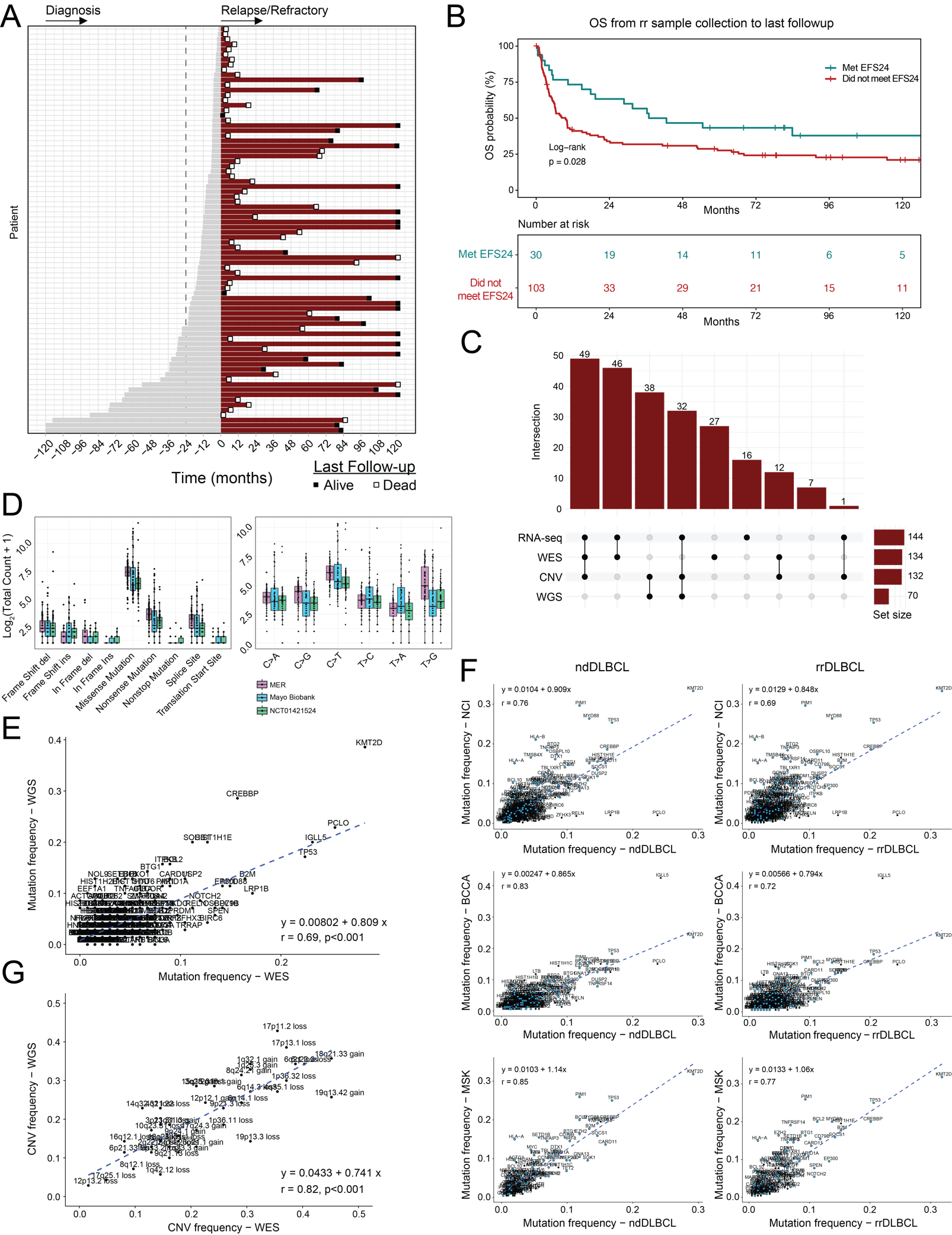
**

**Figure S2:**

1. Duration (by months) of DLBCL diagnosis for rrDLBCL patients (n=80) visualized by swimmers plot. Time 0 indicates the time the first available relapsed/refractory tumor sample was biopsied. From left, grey bars indicate duration under frontline consideration, representing time from diagnosis to relapse. Patients are ranked top to bottom in ascending order according to time from diagnosis to relapse. Black dashed line visualizes 24 months from relapse. To right from time 0, maroon bars indicate duration after the first available relapse/refractory tumor sample was biopsied. Points indicate status at time of last follow-up (filled – alive, open - dead).
2. Kaplan-Meier estimation of OS probability from the time of acquisition of the first available relapse sample to time of last follow-up, stratified by EFS24 achieve/fail status of the respective patient under the frontline setting (met EFS24, n=30, teal; did not meet EFS24, n=103, red). Number of patients available for analysis at indicated time points shown in table. P-value determined via Log-rank test.
3. Quantity and type of high-throughput sequencing data (RNA-seq, n=144; WES, n=134; CNV, n=132; WGS, n=70) from the first available rrDLBCL sample visualized by upset plot.
4. Log2(total counts +1) for respective variant call (top) and nucleotide substitution (bottom) in rrDLBCL samples stratified by source cohort (MER, n=73, purple; Mayo Biobank, n=61, blue; NCT01421524, n=70, green). Dots represent individual tumor samples.
5. Mutation frequency of genes with established potential in DLBCL (n=292) in rrDLBCL samples analyzed by WES (n=134) or WGS (n=70). Trendline and equation represent linear fit. Pearson’s coefficient (r) was calculated for concordance.
6. Mutation frequency of genes with established potential in DLBCL (n=292) in all ndDLBCL (n=404) and all rrDLBCL (n=204) samples compared to publicly available datasets of ndDLBCL: NCI (n=489), BCCA (n=153), and MSK-IMPACT (n=220). Trendline and equation represent linear fit for all genes (n=292). Pearson’s coefficient (r) was calculated for concordance between cohorts for LymphGen genes only (n=125). LymphGen genes represented by blue dots.
7. CNV frequency of regions with established potential in DLBCL (n=45) in rrDLBCL samples sequenced by WES (n=62) or WGS (n=70). Trendline and equation represent linear fit. Pearson’s coefficient (r) was calculated for concordance.

**Figure S3**

**
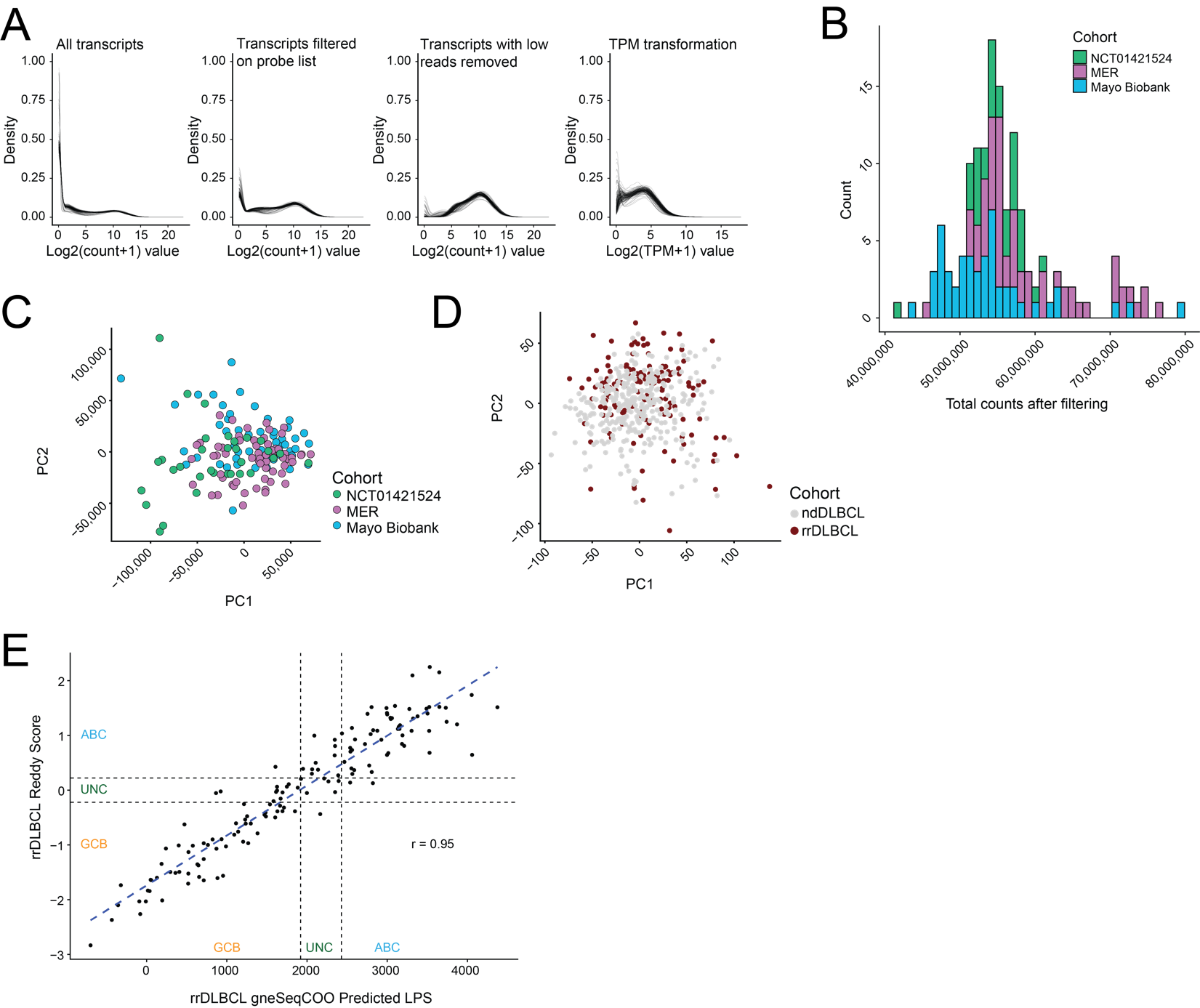
**

**Figure S3:**

1. From left, unfiltered count density plotted as Log2(count+1) for rrDLBCL tumors (n=144) from n=60554 gene IDs, count density plot filtered for probe list (n=26604), count density plot with low read transcripts removed (n=16011), then transformed to transcripts per million (TPM) and plotted as Log2(TPM+1). Lines represent individual tumor samples.
2. Histogram representing distribution of total transcript counts in RNA samples after filtering (NCT01421524, n=32, green; MER, n=61, purple; Mayo Biobank, n=51, blue).
3. Principle component analysis on sample TPMs stratified by cohort (NCT01421524, n=32, green; MER, n=61, purple; Mayo Biobank, n=51, blue). Dots represent individual samples.
4. Principle component analysis on ndDLBCL (n=321; grey) and rrDLBCL (n=144; maroon) sample TPMs. Dots represent individual samples.
5. Scatter plot comparing Reddy COO scores and gneSeqCOO scores for rrDLBCL samples (n=143). GCB, UNC, and ABC thresholds are shown. Pearson’s coefficient (r) is shown.

**Figure S4**

**
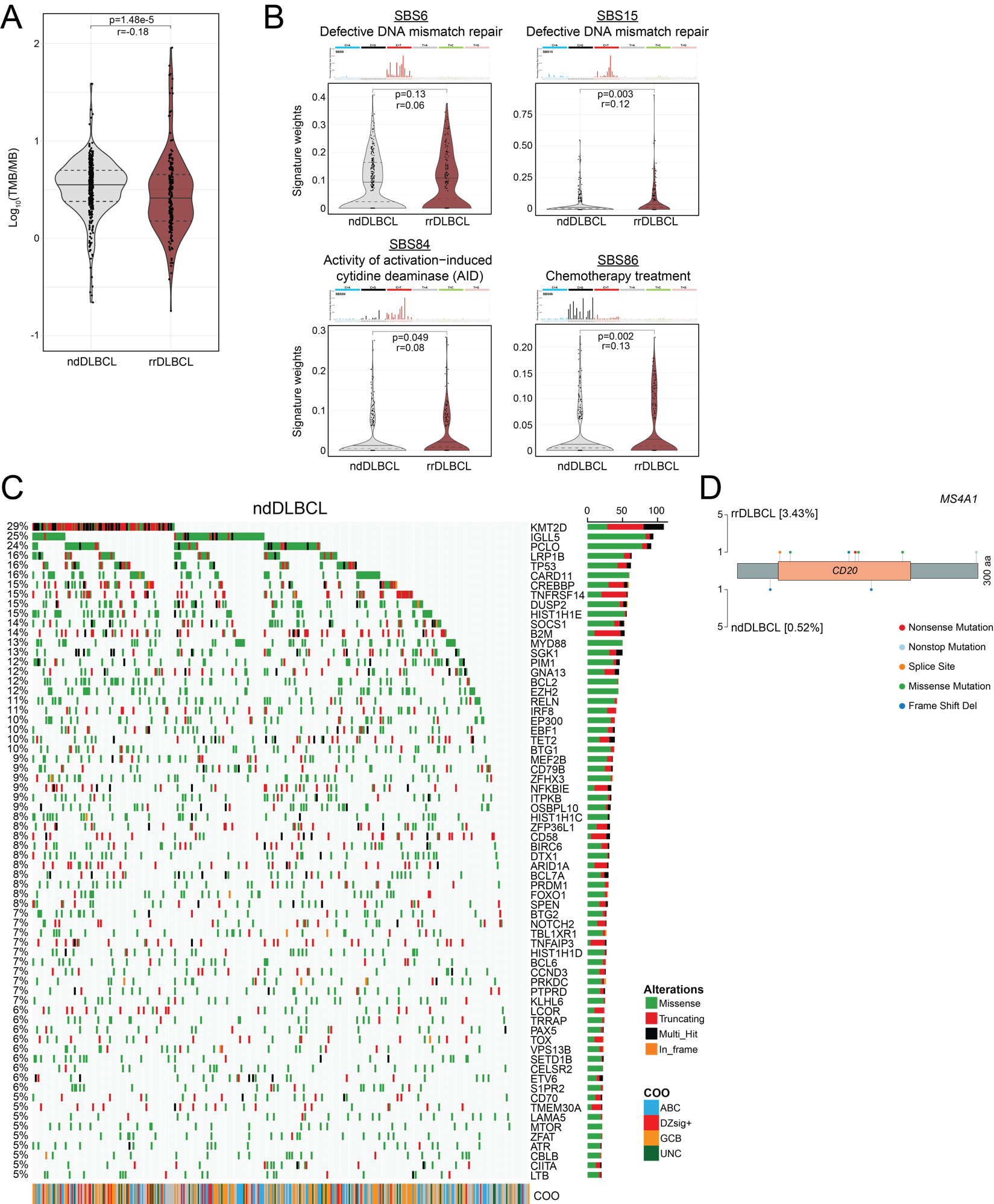
**

**Figure S4:**

1. Comparison of tumor mutation burden (Log10[TMB/Mb]) detected in ndDLBCL (n=382; grey) vs rrDLBCL (n=204; maroon) tumor samples represented as violin plot. Bars indicate median and IQR, dots represent individual tumor samples. P-value determined via Wilcoxon signed-rank test. Effect size (r) is calculated as z statistic divided by square root of the sample size.
2. Mutation signatures in ndDLBCL (n=382) and rrDLBCL (n=204) tumor samples evaluated using single base substitution (SBS) signature patterns described in the COSMIC database (top panel). P-value determined using the Wilcoxon signed-rank test. Effect size (r) is calculated as z statistic divided by square root of the sample size.
3. Oncoplot representing recurrent non-silent somatic single variants or insertions/deletions detected from WES in ndDLBCL tumors (n=404). Predicted functional consequence represented by color (missense, green; truncating, red; in-frame, orange; multi-hit, black). Bar plot to the right represents the sum of patients with a mutation in the respective gene. Genes depicted were filtered for predicted lymphoma driving genes (Supplemental Table 3).
4. Lollipop plot displaying *MS4A1* mutations in ndDLBCL (n=382; bottom panel) and rrDLBCL (n=204; top panel) samples. Vertical displacement represents the number of cases with mutations observed at that position.

**Figure S5**

**
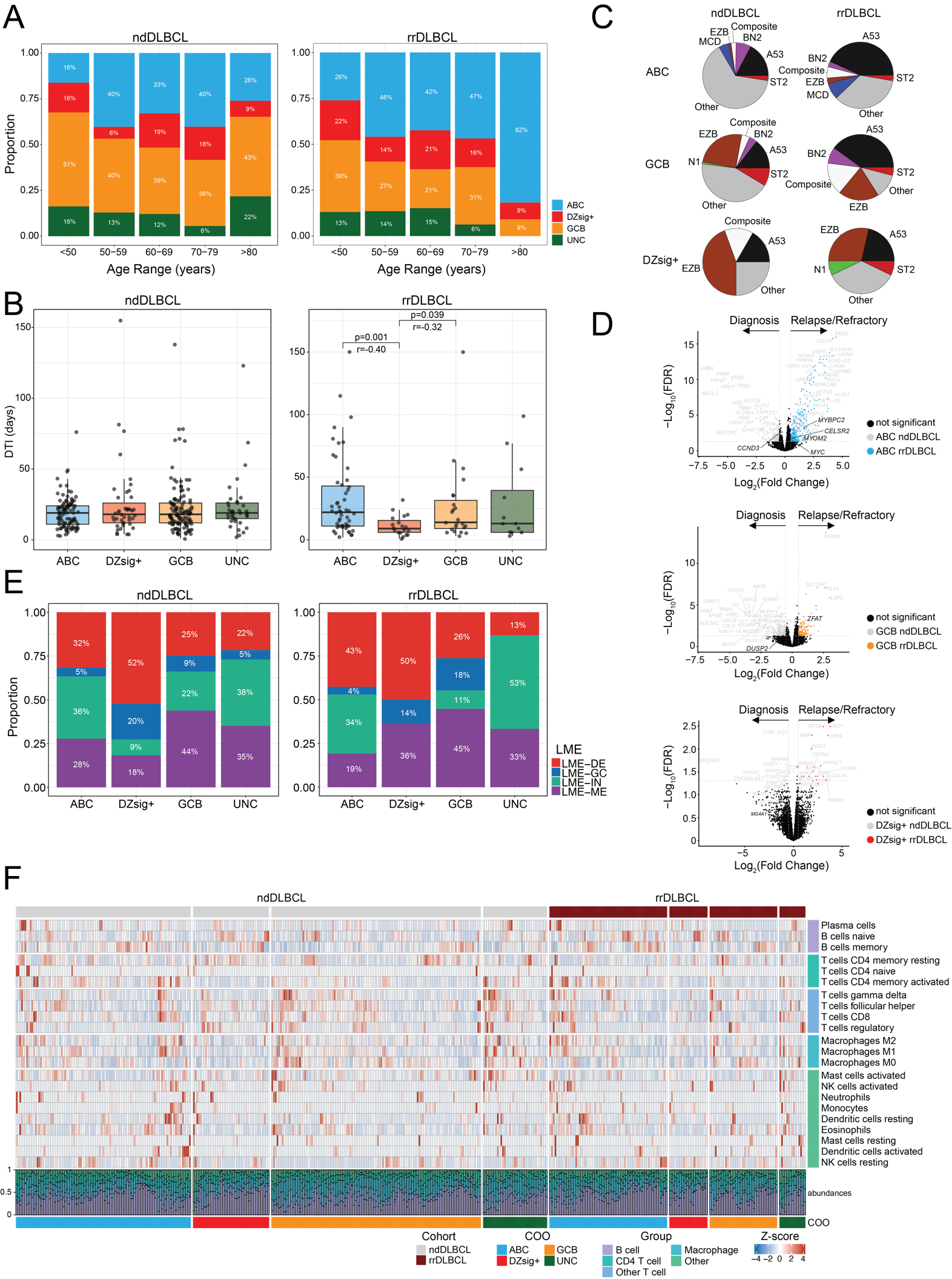
**

**Figure S5:**

1. Proportion of COO among bins grouping range of age of initial diagnosis among patients evaluated with ndDLBCL (<50, n=37; 50-59, n=62, 60-69, n=91; 70-79, n=72; >80, n=23) and rrDLBCL (<50, n=23; 50-59, n=37, 60-69, n=33; 70-79, n=32; >80, n=11) tumors. ABC – blue, DZsig+ – red, GCB – orange, Unclassified (UNC) – green.
2. Comparison of COO and diagnosis-to-treatment interval (DTI) during the diagnostic phase of their disease for patients evaluated with ndDLBCL (ABC, n=101; DZsig+, n=44; GCB, n=121; UNC, n=37) and rrDLBCL (ABC, n=51; DZsig+, n=19; GCB, n=23; UNC, n=12) tumors. Dots represent individual patients. ABC – blue, DZsig+ – red, GCB – orange, Unclassified (UNC) – green.
3. LymphGen classification in ndDLBCL (ABC, n=81; GCB, n=104; DZsig+, n=36) and rrDLBCL (ABC, n=37; GCB, n=25; DZsig+, n=14) tumor samples by COO.
4. Differential gene expression analysis from bulk RNA-sequencing in rrDLBCL and ndDLBCL tumor samples by COO subtype (ABC – n=101 nd, n=68 rr; GCB – n=121 nd, n=39 rr; DZsig+ – n=44 nd, n=22 rr). Genes with significant enrichment (FDR <0.05, |Log2FC| >0.5) in rrDLBCL or ndDLBCL tumors are indicated by color. In ABC and GCB plots, genes with -Log(FDR)>15 & |Log2FC|>2.5 are labeled in grey, while lymphoma driver genes are labeled in black. All genes with enrichment are labeled in DZsig+ plot, including *MS4A1*.
5. Proportion of LME signature classification among COO bins in ndDLBCL (ABC, n=101; DZsig+, n=44; GCB, n=121; UNC, n=37) and rrDLBCL (ABC, n=68; DZsig+, n=22; GCB, n=38; UNC, n=15) tumor samples. LME-DE – red, LME-GC – blue, LME-IN – teal, LME-ME – purple.
6. Cell type abundance of the associated lymphoma microenvironment inferred from gene expression analysis using the CIBERSORTx tool in ndDLBCL (n=303, top grey bar) and rrDLBCL (n=144, top maroon bar) tumor samples. Cell types are grouped by rows (right bars), columns are grouped by COO annotation (bottom bars).
